# Risk Factors for Acquired Rifamycin and Isoniazid Resistance: A Systematic Review and Meta-Analysis

**DOI:** 10.1371/journal.pone.0139017

**Published:** 2015-09-25

**Authors:** Neesha Rockwood, Leila H. Abdullahi, Robert J. Wilkinson, Graeme Meintjes

**Affiliations:** 1 Department of Medicine, Imperial College, London W2 1PG, United Kingdom; 2 Clinical Infectious Diseases Research Initiative, Institute of Infectious Disease and Molecular Medicine, University of Cape Town, Cape Town, South Africa; 3 Francis Crick Institute Mill Hill Laboratory, London, United Kingdom; 4 Department of Medicine, University of Cape Town, Cape Town, South Africa; 5 Vaccines for Africa Initiative, Institute of Infectious Disease and Molecular Medicine, University of Cape Town, Cape Town, South Africa; Indian Institute of Science, INDIA

## Abstract

**Background:**

Studies looking at acquired drug resistance (ADR) are diverse with respect to geographical distribution, HIV co-infection rates, retreatment status and programmatic factors such as regimens administered and directly observed therapy. Our objective was to examine and consolidate evidence from clinical studies of the multifactorial aetiology of acquired rifamycin and/or isoniazid resistance within the scope of a single systematic review. This is important to inform policy and identify key areas for further studies.

**Methods:**

Case-control and cohort studies and randomised controlled trials that reported ADR as an outcome during antitubercular treatment regimens including a rifamycin and examined the association of at least 1 risk factor were included. Post hoc, we carried out random effects Mantel-Haenszel weighted meta-analyses of the impact of 2 key risk factors 1) HIV and 2) baseline drug resistance on the binary outcome of ADR. Heterogeneity was assessed used I^2^ statistic. As a secondary outcome, we calculated median cumulative incidence of ADR, weighted by the sample size of the studies.

**Results:**

Meta-analysis of 15 studies showed increased risk of ADR with baseline mono- or polyresistance (RR 4.85 95% CI 3.26 to 7.23, heterogeneity I^2^ 58%, 95% CI 26 to 76%). Meta-analysis of 8 studies showed that HIV co-infection was associated with increased risk of ADR (RR 3.02, 95% CI 1.28 to 7.11); there was considerable heterogeneity amongst these studies (I^2^ 81%, 95% CI 64 to 90%). Non-adherence, extrapulmonary/disseminated disease and advanced immunosuppression in HIV co-infection were other risk factors noted. The weighted median cumulative incidence of acquired multi drug resistance calculated in 24 studies (assuming whole cohort as denominator, regardless of follow up DST) was 0.1% (5^th^ to 95^th^ percentile 0.07 to 3.2%).

**Conclusion:**

Baseline drug resistance and HIV co-infection were significant risk factors for ADR. There was a trend of positive association with non-adherence which is likely to contribute to the outcome of ADR. The multifactorial aetiology of ADR in a programmatic setting should be further evaluated via appropriately designed studies.

## Introduction

Resistance to both first line antitubercular drugs rifampicin (of the rifamycin drug class) and isoniazid (multi drug resistant tuberculosis (MDR TB)) is an increasing global health problem. The World Health Organisation (WHO) estimates there were 450,000 cases of MDR TB with 170,000 deaths in 2012 [1]. Cure and completion rates are lower than for drug susceptible TB, with higher mortality rates [2] and there is huge cost to health systems. Whilst transmitted drug resistance has been highlighted as important in fuelling the spread of the epidemic, a better understanding of what factors contribute to the initial emergence of resistance is needed to inform policy. Acquired drug resistance (ADR) is the development, fixation and amplification of mutations conferring resistance under drug pressure during treatment. Verification of true ADR requires ruling out initial dual mixed infection or subsequent exogenous re-infection with a drug resistant strain of *M*. *tuberculosis* (MTB).

ADR has been recognised since chemotherapy was first discovered. The early emergence of ADR with streptomycin monotherapy, heralded the need for multidrug regimens to achieve cure and prevent further accumulation of resistance. The inclusion of rifampicin and pyrazinamide in TB regimens since the 1970s led to shortening of TB regimens from 2 years to 6 months. The rate of stochastic acquired drug resistance has been calculated to be in the order of 2.25 x 10^10^ mutations per bacterium per generation for rifampicin and 2.56 x 10^8^ mutations per bacterium per generation for isoniazid [3] within the human host. Upon the background of this natural evolution of resistance, programmatic factors such as problems in maintaining drug supplies and ensuring patient adherence and treatment completion have remained and contributed to the global MDR epidemic through creating the selective pressure necessary for ADR to emerge.

A recently published study of ADR in a hollow fibre model system has questioned the conventional notion that poor adherence accounts for the majority of ADR [4]. Several plausible explanations as to how HIV could predispose to ADR have been proposed including malabsorption of antitubercular drugs [5] and host immunosuppression leading to tolerance of strain-specific polymorphisms in the pathway to drug resistance [6]. However, whether HIV is indeed a risk factor for ADR remains to be clarified. The objective of this review was to consolidate evidence from studies that examined any risk factors for acquired rifamycin and/or isoniazid resistance in patients undergoing antitubercular therapy containing a rifamycin at least during the intensive phase. After conducting the systematic review, a post-hoc decision was taken to carry out 2 separate meta-analyses focused on: 1) HIV infection 2) baseline drug resistance as risk factors for the binary outcome of ADR.

## Methods

We followed the Preferred Reporting Items for Systematic Reviews and Meta-Analyses (PRISMA) guidelines. We registered the review in PROSPERO (crd.york.ac.uk CRD42014003856).

### Selection criteria

We included case-control and cohort studies and randomised controlled trials (RCTs) reporting ADR as either a primary or secondary adverse outcome. To be included, studies had to examine the association of at least 1 risk factor with ADR. Also, patients of any age needed to be on regimens of at least 6 months’ duration which contained rifamycin at least in the intensive phase. We excluded studies that defined ADR as cases of baseline resistance in patients undergoing retreatment for TB. Studies that reported no cases of ADR were excluded. We did not limit our case definition of ADR to studies that had ruled out exogenous re-infection or initial dual mixed infection with different strains using genotypic methods. However, where the data was available, we excluded cases identified as exogenous re-infection via genotyping. Although we collected data on baseline drug sensitivities, the performance of drug sensitivity testing (DST) at baseline in the entire cohort was not required for inclusion. This allowed for inclusion of studies from settings where baseline DST was not routinely performed, but our analyses focussed on those patients in the cohort who did have baseline DST. ADR was defined as identification of new resistance (compared with a baseline isolate of known DST) to rifamycin and/or isoniazid which was made after minimum of 2 weeks on TB treatment or after completion of TB treatment.

### Search strategy

Searches were run in Pubmed/MEDLINE, EMBASE, Cochrane Library, Web of Science, Biosis previews and the Trip Database from 1950 to January 2014. In Pubmed, filters were used to select the following languages: Chinese; English; Italian; Russian; Spanish; French. Our keywords were ‘tuberculosis’ or ‘*Mycobacterium tuberculosis’* AND ‘acquired drug resistance’ OR ‘amplified drug resistance.’ We hand-searched reference lists of reviews and eligible papers for other relevant articles in English.

### Study selection, data extraction and quality assessment

Two reviewers (NR, GM) independently assessed the titles and abstracts of studies from the searches based on pre-specified eligibility criteria. If it was unclear from the abstract whether inclusion criteria were met, the full article was reviewed. Any uncertainty or disagreement about eligibility was resolved through discussion.

The two reviewers then independently extracted data using a structured data extraction form. Any disagreements were discussed. In cases of missing or incomplete information authors were contacted. Critical appraisal tools, developed in the Critical Appraisal Skills Programme (CASP) for judging methodological quality of RCTs, cohort and case control studies, were amalgamated and used to judge methodological quality [7].

### Data synthesis

Risk factors for ADR were tabulated for all studies. If univariate or multivariate analyses were performed, then only if there was a significant association with ADR was the factor categorised as ‘risk factor for ADR’. If no statistical analysis was performed but a risk factor for ADR was described in the study, it was reported as per trend noted. Random effects meta-analyses with Mantel-Haenszel weighting were performed for the covariates baseline drug resistance and HIV co-infection for the binary outcome of ADR using the Cochrane Collaboration Review Manager Version 5.3 statistical software. We calculated risk ratios (RR) and their corresponding 95% confidence intervals (CI) and p-values. Heterogeneity between studies was assessed by calculating the I^2^ statistic and its corresponding 95% CI using Stata version 13.1. As a secondary outcome, the cumulative incidence of acquired isoniazid, rifamycin and MDR was reported for individual studies. Patients with known baseline MDR were excluded from calculations. When feasible, the incidence of ADR was calculated using the following denominators: 1) as a proportion of the whole cohort, 2) as a proportion of those with follow up DST, 3) as a proportion of those with baseline pan-susceptibility, 4) as a proportion of those with baseline monoresistance and 5) as a proportion of those with baseline polyresistance. The median cumulative acquired isoniazid, acquired rifamycin and acquired MDR incidence across all studies that reported these was also calculated, weighted by the overall sample size of each study.

## Results

### Study selection and assessment

**Fig 1:**
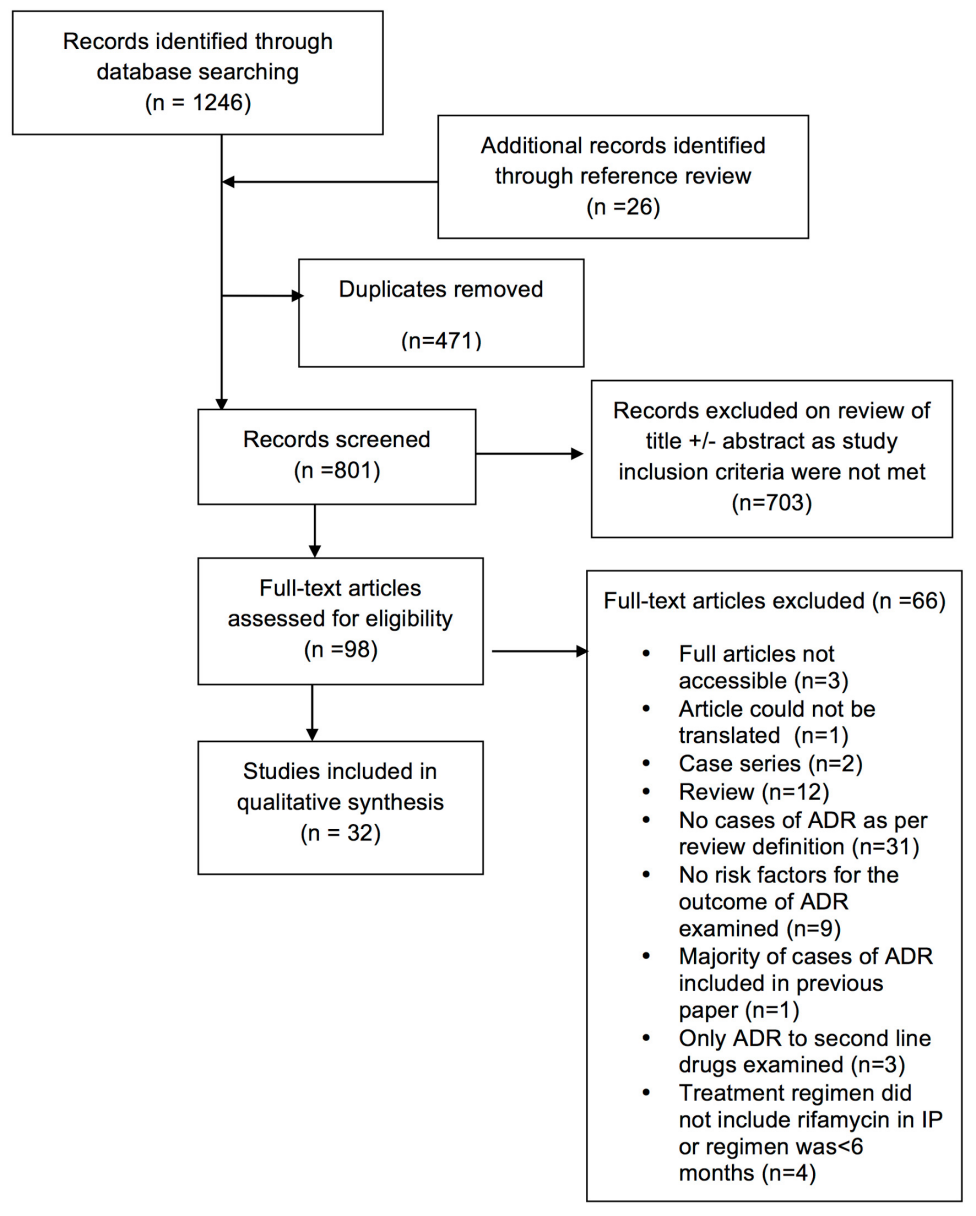
Summary of literature search and study selection.

We identified 798 citations through the electronic database searches: 703 were excluded after abstract review. Another 26 studies were identified through reference review. One hundred full text articles were examined and 32 deemed eligible (6 RCTs, 8 prospective cohort, 15 retrospective cohort and 3 case control studies) (Fig 1).

Table 1, S1 and S2 Tables provide detailed break down and aggregate data of studies included in the review. Certain studies restricted inclusion to specific populations: those with HIV co-infection (n = 5) [8–10], those incarcerated (n = 1) [11], those with silicotuberculosis (n = 1) [12], those with isoniazid monoresistance (n = 1) [13] and retreatment patients (n = 2) [14–15]. S3 Table provides an appraisal of study quality [7]. Loss to follow up was not noted to be significant (pre-defined threshold 20%) in any study. We assessed that in all selected RCTs, treatment effect was measured precisely. We assessed in 20/22 cohort studies, exposure was accurately measured to minimise bias. As illustrated in Tables 2–5, only a proportion of individuals included as the ‘whole cohort’ at baseline had follow up DST as per criteria detailed in S1 Table. These criteria ranged from being performed at a regular monthly interval in all culture positive isolates; to those who were smear/culture positive at 2 and 5–6 months; to being only performed in cases of suspected failure/relapse. In some cases, this may have compromised accuracy of measurement of outcome.

**Table 1:** Characteristics of included studies including HIV co-infection, proportion receiving retreatment, treatment regimen, whether treatment was self-administered or directly observed and use of genotyping.

Reference	Study location and year	HIV prevalence	Retreatment (ReRx)	Regimen	DOT	Genotyping carried out in a proportion of available isolates
RCTs						
Algerian Working Group/British MRC 1991 Tubercle [16]	Algeria Oct 1981-Dec1983	0%	Not specified	IP: Regimen 1) 2(HRSZ_7_) Regimen 2) 2(HREZ_7_) CP: Regimen 1) 4(HR_7_) Regimen 2) 2(HR_7_)4(H_7_)	DOT in IP (whilst on streptomycin)	No
Hong Kong TB Research Centre Madras/BMRC Am Rev Resp Disease 1991 [12]	Hong Kong Dec 1980–Dec 1985	Not specified	Not specified	Regimen 1) 6(RHSZ) Regimen 2) 8(RHSZ)_3_(E was added for first 3 months if retreatment patient)	100%	No
Lienhardt JAMA 2011 [17]	Algeria, Colombia, Guinea, Vietnam, Peru,Mozambique, Tanzania, Bolivia 2003–2008	6.6%	0%	IP: Regimen 1) 2(RHEZ_7_) as FDC Regimen 2) 2(RHEZ_7_) as single drugs CP: 4(RH_3_)	100%	Yes Spoligo and MIRU-VNTR
Swaminathan AJRCCM 2010 [10]	Chenai, India Feb '01–Sep '05	100%	0%	IP: Regimen 1) 2(RHEZ_3_) Regimen 2) 2(RHEZ_3_) CP: Regimen 1) 4(RH_3_) Regimen 2) 7(RH_3_)	DOT was given during IP. 1/3 doses was given as DOT during CP	Yes IS6110, MIRU-VNTR, Spoligo
TB Research Centre IJTLD 1997 [18]	Chennainot specified	Not specified	Not specified	IP: Regimen 1) 2(HREZ_7_) Regimen 2) 2(HREZ_2_) Regimen 3) 2(HRZ_2_)CP: Regimen 1) 6(HE_7_) Regimen 2) 4(HRE_2_) Regimen 3) 4(HR_2_)	Regimen 1 was fully unsupervised. Regimen 2 and 3 were either fully or partially supervised.	No
Vernon Lancet 1999 [8]	USAApr 1995–early 1997	100%	47.5%	IP: 2(RHEZ_7/3/2_) CP: Regimen 1) 4(rifapentine/H_1_) Regimen 2) 4(RH_2_)	100%	YesIS6110
Prospective cohorts						
Aung, IJTLD 2012 [19]*operational study with randomisation	BangladeshJan '06–Jun '07	Not specified	0%	IP: 2(3) RHEZ_7_ CP: 4(HR_3_)	100%	Yes Sequencing of core region of *rpoB* gene
Burman AJRCCM 2006 [9]	New York City, USA Dec 1998–Mar 2002	100%	Not specified	IP: First 2 weeks: (RHEZ_7_) Next 6 weeks: (RHEZ_5_) or (RHEZ_3_) or (RHEZ_2_) (78% received rifampin in IP) CP: 4-7(RH_2_) R = rifabutin	100%	Yes Sequencing of core region of *rpoB* gene
Cox, Clin Infect Dis 2007 [20]	Karakalpakstan, Uzbekistan and Dashoguz, Turkmenistan Jul 2001–Mar 2002	Not specified	45%	IP: New 2(HREZ_7_) ReRx 2(SRHEZ7),1(RHEZ_7_) CP: New: 4(HR_3_) ReRx:5(HRE_3_)	DOT during IP	Yes RFLP of IS6110 and spoligo
El Sahly, J of Infect, 2006 [21]	Houston, USA 1995–2001	18.1%	6.3%	*Not specified	Not specified	Yes RFLP of IS6110 and spoligo
Murray SAMJ 2000 [22]	Goldmines in Gauteng, South Africa, 1995	49%	27%	IP: 2RHZE CP: 4RH	DOT if smear+	No
Nettles, Clin Infect Dis 2004 [23]	Baltimore, USA Jan '93–Dec '01	27%	Not specified	IP: 2wks (RHEZ_7_) 6wks (RHEZ_2_) Rifampicin or rifabutin CP: (RHEZ_2_) Rifampicin or rifabutin, duration individualised	100%	YesRFLP of IS6110
Pasipanodya, J Inf Dis 2013 [24]	Western Cape, South Africa	10%	64%	IP: New 2(HREZ_7_) ReRx 2(SRHEZ7),1(RHEZ_7_) CP: New: 4(HR_3_) ReRx:5(HRE_3_)	DOT during IP	No
Temple Clin Infect Dis 2008 [14]	Kampala, Uganda Jul 2003–Nov 2006	48%	100%	IP: 1(SRHEZ_7_) 2(RHEZ_7_) CP: 5(RHE_7_)	DOT in IP (hospitalised)	Yes RFLP of IS6110
Retrospective cohorts						
Chien, JAC 2013 [25]	Taiwan 2005 to 2011	0%	Not specified	WHO recommendations IP: New 2(HREZ_7_) ReRx 2(SRHEZ7),1(RHEZ_7_)CP: New 4(HR_3_) ReRx 5(HRE_3_)	57% received DOTS	No
Driver, Clin Infect Dis, 2001 [26]	New York City Jan 1993–Jun 1996	33%, (unknown 36%)	0%	IP: Regimen 1) Regimen 2(RHZ_7_) Regimen 2) 2(RHZ_7_) Regimen 3) IP with < 8weeks of Z CP: Regimen 1) 4(RH_7_) Regimen 2) 6(HE_7_) Regimen 3) 7(RH_7_)	DOT median 21 weeks	Yes RFLP of IS6110
Gelmanova, Bull WHO, 2007 [27]	Tomsk, Siberia Jan 2001–Dec2001	1%	Not specified	WHO recommendations IP: New) 2(HREZ_7_) ReRx): 2(SRHEZ7),1(RHEZ_7_)CP: New: 4(HR_3_) ReRx:5(HRE_3_)	DOT in inpatient, outpatient and home care setting. Small proportion self-administered therapy	No
Jasmer, AJRCCM, 2004 [28]	San Francisco, United States 1998 to 2000	13%	9%	*Not specified	DOT (n = 149) and SAT (n = 223)	No
Kim BMC ID 2008 [13]	Seoul, Korea Jul 2001–Jun 2005.	36%	Not specified	IP: 2(RHEZ_7_) CP: 33% 4(REZ_7_) 54% 10(RE_7_) 13% 7(RE_7_)	Not specified	No
Li CID 2005 [29]	New York City Jan 1997–Dec 2000	Not specified	28%	IP: Variable rifampin or rifabutin-based regimen, daily or intermittent dose (2/wk or 3/wk) for 2 months CP: rifampin or rifabutin regimen given x2 or 3/wk for 4–6, 7–10 or >10 months	Not specified	YesRFLP of IS6110 and spoligo
Matthys, PLoS ONE, 2009 [11]	Mariinsk, Siberia, Russia 1997 to 1998	None at entry into prison	65%	IP: 2(SRHEZ_7_),1(RHEZ_7_) CP: 5(RHE_7_)	100%	Yes RFLP of IS6110
Moulding IJTLD 2004 [30]	Los Angeles, US Jun 1985–Jul 1992	Cohort known or presumed to be HIV negative	Not specified	IP: HR and Z or E or ZE (duration and frequency not specified) CP: HR (duration and frequency not specified)	Not specified	No
Porco CID 2012 [31]	California, USA Jan 1994–Dec 2006	7.5%	Not specified	*Not specified	100%	No
Quy IJTLD 2003 [32]	Ho Chi Minh City, Vietnam Aug 1996–Jul 1998	Not specified	0%	IP: New: 2(SHRZ_7_) ReRx 2(SRHEZ_7_),1(RHEZ_7_) CP: New: 6(HE_7_) ReRx:5(HRE_3_)	100%	YesRFLP of IS6110
Seung CID 2004 [33]	Tomsk, Siberia Nov 1996–Dec 2000	Not specified	0%	IP: 2(HREZ_7_) In some cases S was given instead of E CP: 4(HR_7_)	DOT programme IP- hospitalised CP- outpatient	No
Spellman 1988 AIDS [34]	Miami and New York, USA Jan '88–Dec '95	12.8	5.2%	*Not specified	100%	No
Weis, NEJM 1994 [35] #Although presented under cohort studies, this may also be classified as an ecological study	United States 1980 to 1992	58 amongst 485 those tested from 1987 (12%)	Not specified	IP: 1980 to 1986 included HRE. 1986 to 1992 included HRZ +/- E or injectable CP: Not specified	Until 1986 not DOT, from 1986 90.5% received DOT	No
Yoshiyama IJTLD 2004 [15]	Chiang Rai, Thailand May 1996–Dec 2000	31%	100% of re-registered cohort	IP: 2(SRHEZ_7_),1(RHEZ_7_) CP: 5(RHE_7_)	DOT introduced in 1996	Yes RFLP of IS6110
Yuen, PLoSONE 2013 [36]	United States2004 to 2011	Positive 7% Negative 67.5% Unknown 25.5%	0%	*Not specified	DOT only 61%, DOT + SAT 37%, SAT only 2%	No
Case controls						
Bradford Lancet 1996 [37]	San Fransciso, USA Jan '85–Dec '94	Cases 79% Controls 27%	Cases 14% Controls 14%	*Not specified	Not specified	Yes RFLP of IS6110
Munsiff, Clin Infect Dis 1997 [38]	New York City, USA 93–94	100%	Not specified	IP: Regimen contained RHZ (+/-E), dosing regimen not specified CP: *Not specified	Cases: 24% received DOT Controls: 31% received DOT	No
Weiner CID 2005 [39]	New York City Dec 1998–Mar 2002	100%	Not specified	IP: First 2 weeks: (RHEZ_7_) Next 6 weeks: (RHEZ_5_) or (RHEZ_3_) or (RHEZ_2_) CP: 9(RH_2_)	100%	Yes Sequencing of core region of *rpoB* gene

Abbreviations: IP intensive phase CP continuation phase R rifampin H isoniazid E ethambutol Z pyrazinamide S streptomycin Rx treatment wk week DOT directly observed therapy SAT self-administered therapy X(RHEZ_Y_) X = number of months on regimen y = number of days/week on regimen ARR acquired rifamycin resistance Spoligo Spoligotyping MIRU-VNTR (mycobacterial interspersed repetitive unit-variable- number tandem repeat) typing RFLP of IS6110 restriction fragment length polymorphism of the IS6110 insertion element

*individualised treatment as per Centre of Disease Control, USA guidelines http://www.cdc.gov/mmwr/pdf/rr/rr5211.pdf.

Whilst treatment regimens were not explicitly stated in 6 (19%) of studies, these all included treatment with a rifamycin during intensive phase and were of minimum 6 months duration.

**Table 2:** RCTS- ADR and associated risk factors.

Reference	Cohort description and numbers	Acquired isoniazid resistance (%)	Acquired rifamycin resistance (%)	Acquired MDR TB (%)	Risk factors associated with acquired drug resistance (ADR)
Algerian Working Group/British MRC 1991 Tubercle [16]	Whole cohort n = 2218 Known baseline drug sensitivity n = 2071 Follow up drug sensitivity n = 1415	WC = 1/2071 (0.05) WCFU = 1/1415 (0.07) PS = 1/1376 (0.07) MR_(S)_ = 0/40 (0)	WC = 4/2071 (0.19) WCFU = 4/1415 (0.28) PS = 1/1376 (0.07) MR_(H/S)_ = 0/61 (0) PR _(H+S)_ = 3/34 (8.8)	WC = 4/2071 (0.19)WCFU = 4/1415 (0.28) PS = 1/1376 (0.07) MR_(H /S)_ = 0/50 PR_(H+S)_ = 3/33 (9)	-Rifampicin in regimen only during intensive phase -Baseline resistance to INH and STREP
Hong Kong TB Research Centre Madras/BMRC Am Rev Resp Disease 1991 [12]	Whole cohort n = 145 Culture proven TB with known baseline sensitivity n = 127	WCFU 2/127 (1.6) PS = 2/91 (2.2) MR_(S/R)_ = 0/13 (0)	WCFU 5/127 (3.9) PS 1/91 (1.1) MR_(H/S)_ = 1/22 (4.5) PR = 3/9 (33)	WC = 4/127 (3.1) PS = 1/91 (1.1) MR_(H/S/R)_ = 1/25 (4) PR_(H+S)_ = 3/9 (33)	-Baseline drug resistance
Lienhardt JAMA 2011 [17]	Culture confirmed smear +ve new TB patients either pan-susceptible or INH monoresistant n = 1170	WCFU 1/1170 (0.09) PS 1/1005 (0.1)	WCFU 1/1170 (0.09) PS 0/1005 (0) MR_(H)_ 1/127 (0.79)	WCFU 1/1170 (0.09) PS 0/1005 (0) MR_(H)_ 1/127 (0.79)	-None of the factors analysed were associated (see S4 Table)
Swaminathan AJRCCM 2010 [10]	New TB cases (baseline MDR excluded) n = 327 Culture confirmed with DST and results at end of treatment n = 212	WC 7/327 (2) WCFU 7/212 (3.3) PS 7/194 (3.6)^¥^	WC 20/327 (6.1) WCFU 20/212 (9.4) PS 11/194 (5.7)^¥^	WC 17/327 (5.2) WCFU 17/212(8.0) PS 11/194 (5.7)^¥^	-Lower median CD4 lymphocyte count (p 0.054)- Higher median HIV VL (p 0.009) -Non-adherence (adherence <90%) (p 0.000) -Baseline isoniazid resistance (OR 8.43, p 0.002)
TB Research Centre IJTLD 1997 [18]	Smear +ve TB n = 1203, Followed up post end of treatment and included in relapse analyses n = 777	WC 22/1053 (2.1) PS 22/825 (2.7) MR_(R)_ 1/1 (100)	WC 26/1053 (2.4) PS 3/825 (0.3) MR_(H)_ 23/227 (10)	WC 26/1053 (2.4) PS 1/825 (0.1) MR_(H/R)_ 23/228(10)	-Lack of ethambutol in a twice weekly regimen -Baseline drug resistance
Vernon Lancet 1999 [8]	Culture confirmed drug sensitive TB, HIV co-infected n = 61	Not specified	WCFU 4/61 (6.6) PS 4/61 (6.6)^¥^	Not specified	-Once-weekly isoniazid/rifapentine (p 0.05) Baseline CD4 (p 0.02) -Age (p 0.04) -Extrapulmonary + pulmonary disease (p 0.03)—Use of antifungal azoles (p 0.006)

Abbreviations: WC whole cohort denominator known DST; WCFU denominator f/u DST; PS denominator initial pan-sensitivity; MR denominator initial monoresistance; PR denominator initial polyresistance. H isoniazid S Streptomycin R rifampicin DOT directly observed therapy NTM non-tuberculous mycobacteria MDR multidrug resistant ART antiretroviral therapy BMI body mass index INH isoniazid PZA pyrazinamide.

^¥^ADR data presented is not stratified by baseline monoresistance and polyresistance as this information cannot be ascertained from the paper.

**Table 3:** Prospective cohorts- ADR and associated risk factors.

Reference	Cohort description and numbers	Acquired isoniazid resistance (%)	Acquired rifamycin resistance (%)	Acquired MDR TB (%)	Risk factors associated with ADR
Aung, IJTLD 2012 [19] *operational study with randomisation	Patients who were smear -ve at 2mth and whose IP was not extended n = 12967 Patients who were smear +ve at 2mth and whose IP was extended by 1 mth n = 1871 Patients who were smear +ve at 2mth and whose IP was not extended n = 1870 Smear defined relapses/failures n = 595	Not specified	WC 16/16708 (0.09) WCFU 16/595 (2.7)	WC 12/16708 (0.07) WCFU 12/595 (2)	None of the factors analysed were associated (see S4 Table)
Burman AJRCCM 2006 [9]	Culture confirmed TB, HIV co-infection and on intermittent rifabutin* based regimen n = 169*78% initially received a median of 33.5 days of rifampin based therapy during IP	Not specified	WC = 8/169 (4.7)	WC = 1/169 (0.59)	-Lack of use of ART in first 2 months TB treatment (p = 0.05)—Lower CD4 lymphocyte count at diagnosis (p = 0.001)
Cox, Clin Infect Dis 2007 [20]	Smear +ve TB (baseline MDR patients and mixed infection excluded) n = 314, smear +ve with identical spoligotype as baseline at end of IP or 2 months into CP n = 62	WCFU 1/314 (0.3) PS 1/177 (0.6) MR_(R/S/Z/E)_ 0/51 (0)	WCFU 11/314 (3.5) PS 1/177 (0.6) MR_(I/S/Z/E)_ 0/71 (0) PR_(H+S or I+S+E or I+S+E+Z)_ 10/65 (15.3)	WCFU 11/314 (3.5) PS 1/177 (0.6) MR_(I/S/R/Z/E)_ 0/72 (0) PR_(I+S or I+S+E or I+S+E+Z)_ 10/65 (15.3)	-Baseline polyresistance (p<0.05)—Beijing genotype in polyresistant strain (11 out of 28 polyresistant Beijing strains amplified their resistance, compared with none of the 27 non-Beijing strains)
El Sahly, J Infect, 2006 [21]	Pan-susceptible TB n = 1977	PS 9/1977 (0.45)	PS 7/1977 (0.3)	PS 1/1977 (0.05)	-HIV positivity (aOR 5.52 95% CI 1.55–19.68)—Asian ethnicity (aOR 16.74 95% CI 3.8–73.72)—smear positive (aOR 4.76 95% CI 1.42–15.96)—Disseminated TB (with pleural effusion) (aOR 9.22 95% CI 2.82–30.17) aOR adjusted odds ratio
Murray SAMJ 2000 [22]	Culture confirmed drug sensitive/monoresistant TB cases (MDR cases excluded) n = 400	WCFU 6/400 (0.15) PS 6/350 (0.17)^¥^	WCFU 6/400 (0.15) PS 3/350 (0.9) ^¥^	WCFU 6/400 (0.5) PS 3/350 (0.9)^¥^	-Baseline drug resistance
Nettles, Clin Infect Dis 2004 [23]	Culture confirmed TB (excluding drug resistance, no DOT, alternative regimen, loss to follow up, death) n = 407	Not specified	WCFU 3/407 (0.7)	Not specified	-HIV co-infection (p = 0.02) -Lower baseline median CD4 lymphocyte count (p = 0.02)
Pasipanodya, J Inf Dis 2013 [24]	Smear or culture confirmed drug sensitive TB n = 142	WCFU 3/142 (2%)	WCFU 1/142 (0.7%)	WCFU (0.7%)	-PK variability including PZA AUC_24_ ≤363 mg*h/L, RIF AUC_24_≤13 mg*h/L, and INH AUC_24_≤52mg*h/L
Temple Clin Inf Dis 2008 [14]	Smear +ve, culture +ve retreatment TB cases with known DST at baseline admitted to hospital (excluding MDR) n = 269, 5 month follow up sputa n = 237	WC 2/269 (0.7) WCFU 2/237 (0.8) PS 2/226 (0.9) MR_(S/R)_ 0/11 (0)PR_(S+Z/S+E/S+Z+E)_ 0/9 (0)	WC 6/269 (2.2) WCFU 6/237 (2.5) PS 3/226 (1.3) MR_(I/S/Z/E)_ 3/38 (10.3) PR_(S+Z/S+E/S+Z+E)_ 0/9 (0)	WC 5/269 (1.9) WCFU 5/237 (2.1) PS 2/226 (0.9) MR 3/31 (9.6) PR 0/9 (0)	-Baseline resistance (HR 10, p = 0.003)

Abbreviations: WC whole cohort denominator known DST; WCFU denominator f/u DST; PS denominator initial pan-sensitivity; MR denominator initial monoresistance; PR denominator initial polyresistance. H isoniazid S Streptomycin R rifampicin E ethambutol Z pyrazinamide, DOT directly observed therapy IP intensive phase CP continuation phase Hb haemoglobin ART antiretroviral therapy BMI body mass index INH isoniazid PZA pyrazinamide RIF rifampin AUC_24_ 24 hr area under the concentration-time curve.

^¥^ADR data presented is not stratified by baseline monoresistance and polyresistance as this information cannot be ascertained from the paper

**Table 4:** Retrospective cohorts- ADR and associated risk factors.

Reference	Cohort description and numbers	Acquired isoniazid resistance (%)	Acquired rifamycin resistance (%)	Acquired MDR TB (%)	Risk factors associated with ADR
Chien, JAC 2013 [25]	Culture confirmed pulmonary TB without HIV co-infection (baseline MDR/XDR excluded) n = 2080	WCFU 108/2080 (5.2)	WCFU 160/2080 (4.7)	WCFU 178/2080 (8.6)	-Age group 45–64 (OR 2.07, p = 0.01) -Smear positivity (OR 2.09, p = 0.01)—Self-administration of treatment/lack of DOT (OR 2.94, p = 0.01)
Driver, Clin Infect Dis, 2001 [26]	Drug sensitive at baseline n = 4571, Known relapses/recurrences at end of at least 6mths Rx n = 123, Known DST at relapse or recurrence n = 95	WCFU 14/4571 (0.3) PS 14/4571 (0.3)	WCFU 21/4571 (0.5) PS 21/4571 (0.5)	WCFU 9/4571 (0.2) PS 9/4571 (0.2)	-HIV co-infection (risk factor for acquired RIF monoresistance)
Gelmanova, Bull WHO, 2007 [27]	Enrolled in DOT n = 260, Culture +ve, with known DST (baseline MDR at excluded) n = 207	Not specified	Not specified	WCFU 15/207 (7.3)	-Substance abuse (HR 2.88, p = 0.04)-Treatment commenced in hospital setting (HR = 6.34, p = 0.02)—Hospitalisation later in treatment (HR = 6.26, p = 0.047) -Self-administration of Rx/lack of DOT (HR 0.25, p = 0.03)
Jasmer, AJRCCM, 2004 [28]	Drug sensitive cases of TB who started treatment with allocated DOT status n = 372, Had f/u cultures as part of post-treatment evaluation n = 330	WC 2/372 (0.5) WCFU 2/330 (0.6) Details of which drugs resistance is acquired to not specified			None of the factors analysed were associated (see S4 Table)
Kim BMC ID 2008 [13]	INH resistant at baseline n = 39	N/A	WCFU 2/39 (5.1) MR_(H)_ 2/39 (5.1%)	WCFU 2/39 (5.1) MR_(H)_ 2/39 (5.1)	-2 vs 3 drugs in continuation phase -Extensive radiological disease -Smear positivity
Li CID 2005 [29]	Confirmed TB with known DST, n = 2861	Not specified	WCFU 10/2861 (0.3)	Not specified	-HIV infection aOR, 5.5; 95% CI, 1.4–21.5) Analysis restricted to CD4 count <100: Rifampicin-based (as opposed to rifabutin) regimens aHR 8.5; 95% CI, 1.03–70.9) Analysis restricted to HIV patients on rifampicin-based regimens: patients received intermittent dosing during IP HR 6.4; 95% CI, 1.1–38.44) aOR adjusted odds ratio aHR adjusted hazards ratio
Matthys, PLoS ONE, 2009 [11]	Admitted and remained inpatient in penitentiary hospital during treatment (baseline MDR excluded) n = 189	WCFU 0/189 (0) PS 0/81 (0) MR_(S/R/E)_ 0/20	WCFU 6/189 (3.2) PS 0/81 (0) MR_(H/S/E)_ 0/46 (0) PR_(H+E/H+S/H+S+E)_ 6/61 (9.8)	WCFU 6/189 (3.2) PS 0/81 (0) MR_(H/S/R/E)_ 0/47 (0) PR_(H+E/H+S/H+S+E)_ 6/61 (9.8)	-Amplification of resistance occurred in 10.7% of those with polyresistance at baseline (vs 3.4% in the whole cohort)
Moulding IJTLD 2004 [30]	Drug sensitive at baseline n = 5337	WC 25/5337 (0.47) Details of which drugs resistance is acquired to not specified			- Separate drug formulation as opposed to fixed dose combination-Private sector management
Porco CID 2012 [31]	Drug sensitive at baseline n = 33725 Repeat DST at follow up n = 1792	WC 52/33725 (0.15) WCFU 52/1792 (2.9) PS 46/30 548 (0.15) MR_(R)_ 6/138 (4.3)	WC 64/33725 (0.19) WCFU 64/1792 (3.5) PS 37/30 548 (0.1) MR_(I)_ 27/3039 (0.9)	WC 49/33725 (0.1) WCFU 49/1792 (2.7) PS 16/30 548 (0.05) MR_(H/R)_ 33/3177 (1)	**Acquired INH resistance**: -Initial rifampicin resistance (aOR 10.3 p<0.001] -HIV infection (aOR = 3.36, p = 0.01)**Acquired rifampicin resistance**:- Age per year (aOR 0.96, p = 0.002)-HIV infection (aOR = 9.39, p<0.001)-Initial resistance to isoniazid (aOR = 11.2, p<0.001)-Cavitatory disease in the absence of DOT (OR = 2.65, p 0.03) **Acquired MDR:**- Initial isoniazid resistance (aOR = 19.2p<0.001)- Initial rifampicin resistance (aOR = 35.9, p<0.001) -HIV infection (aOR = 5.07, p = 0.003) -Cavitatory disease in the absence of DOT (aOR = 2.65, p = 0.04) aOR = adjusted odds ratio
Quy IJTLD 2003 [32]	New smear +ve cases starting TB Rx n = 2901, Culture positive failure and relapse cases with repeat DST and identical ISS610 patterns compared with baseline (baseline MDR excluded)n = 62	WC 3/2901 (0.1)WCFU 3/62 (4.8)^¥^	WC 18/2901 (0.6) WCFU 17/62 (2.7)^¥^	WC 18/2901 (0.6) WCFU 17/62 (2.7)^¥^	-Baseline drug resistance (OR 6.6, 95% CI 1.4–32)
Seung CID 2004 [33]	Enrolled in category 1 treatment regimen n = 2194, Culture positive cases with known DST (baseline MDR cases excluded) n = 1610	WC 19/1610 (1.2) PS 9/1212 (0.7) MR_(R/E/S)_ 6/191 (3.1) PR_(R+E/R+S/R+E+S)_ 2/27 (7.4)	WC 31/1610 (1.9) PS 9/1212 (0.7) MR_(H/E/S)_ 6/212 (2.8) PR_(H+S/H+E/H+S+E)_ 16/153 (10.4)	WC 28/1610 (1.7) PS 8/1212 (0.7) MR 6/225 (2.7) PR 18/180 (10)	-Baseline drug resistance
Spellman 1988 AIDS [34]	TB patients with known DST (baseline MDR cases excluded) n = 739	WC 2/739 (0.3) PS 2/682 (0.3) MR_(S/R/Et)_ 0/23 (0) PR_(S+E/S+Et)_ 0/5 (0)	WC 4/739 (0.5) PS 2/682 (0.3) MR_(H/S/Et)_ 1/42 (2.4) PR_(H+S/H+E/H+Et/H+Et+S/S+E/S+Et)_ 1/14 (7.1)	WC 2/739 (0.3) PS 2/682 (0.3) MR_(H/S/R/Et)_ 1/65 (1.5)PR _H+S/H+E/H+Et/H+Et+S/S+E/S+Et)_ 1 /14 (7.1)	-Baseline drug resistance
Weis, NEJM 1994 [35]	Culture confirmed TB (baseline MDR cases excluded), n = 957	Not specified	Not specified	WC 47/957 (4.9)	-Self-administration of treatment/lack of DOT (p<0.001)
Yoshiyama IJTLD 2004 [15]	Culture positive cases (baseline MDR cases excluded) n = 1871, patients with repeat DST n = 704 Re-registered cases with DST n = 59	WC 5/1871 (0.3) WCFU 5/704 (0.7) PS 1/1634 (0.06) MR_(R)_ 4/43 (0.09)	WC 11/1871 ((0.6) WCFU 11/704 (1.6) PS 4/1634 ((0.2) MR_(H)_ 7/107 (6.5)	WC 12/1871 (0.6) WCFU 12/704 ((1.7) PS 1/1634 (0.06) MR_(H/R)_ 11/150 (7.3)	-Previous treatment failure-Baseline resistance—HIV co-infection
Yuen, PLoSONE 2013 [36]	Culture confirmed new cases with initial DST n = 51,223, Known genotype and follow up DST (n = 3696 for isoniazid, and n = 4005 for rifamycin)	WC 61/51223 (0.1) WCFU 61/3696 (1.7)	WC 50/51223 (0.1)WCFU 50/4005 (1.2)	Not specified	**Acquired INH resistance** -*M*. *bovis* (aPR = = 8.46, 95% CI 2.96–24.14)**Acquired rifamycin resistance**—*M*. *bovis* (aPR = 4.53, 95% CI 1.29–15.90) -Homeless (aPR = 2.21, 95% CI 1.08–4.52)-HIV co-infection (aPR 8.89, 95% CI 4.43–17.85) -Initial isoniazid resistance (aPR 10.37, 95% CI 5.65–19.00)—Extrapulmonary only disease (aPR 2.31, 95% CI 1.17–4.58) -Self-administered therapy/lack of DOT (aPR 2.52, 95% CI (1.01–6.30) -Initial ethambutol resistance (aPR 4.22, 95% CI (1.06–16.76) -Injecting drug use (aPR 4.09, 95% CI 1.66–10.10) -Age 45–64 (aPR 0.46, 95% CI 0.25–0.85),-Age>65 (aPR 0.14, 95% CI 0.03–0.58) aPR = adjusted prevalence ratio

Abbreviations: WC whole cohort denominator known DST; WCFU denominator f/u DST; PS denominator initial pan-sensitivity; MR denominator initial monoresistance; PR denominator initial polyresistance.H isoniazid S Streptomycin R rifampicin E ethambutol Et ethioniamide DOT directly observed therapy IP intensive phase CP continuation phase Hb haemoglobin ART antiretroviral therapy BMI body mass index INH isoniazid PZA pyrazinamide R rifampin E ethambutol.

^¥^ADR data presented is not stratified by baseline monoresistance and polyresistance as this information cannot be ascertained from the paper

**Table 5:** Case-Control studies- ADR and associated risk factors.

Reference	Cohort description and numbers	Acquired isoniazid resistance (%)	Acquired rifamycin resistance (%)	Acquired MDR TB (%)	Risk factors associated with ADR
Bradford Lancet 1996 [37]	Total TB cases reported with known DST n = 2612 Cases: acquired resistance to R,H or E with baseline pan-susceptibility n = 14 Control: baseline pan-susceptibility, no ADR, matched to time of diagnosis as cases n = 56	WC 7/2612 (0.3)	WC 10/2612 (0.3)	WC 3/2612 (0.1)	- White ethnicity (p = 0.015) -Foreign birth (p = 0.007) -Unemployment (p = 0.017)—Self-administration of treatment/lack of DOT (p = 0.045)-ART use (p = 0·014) -Azole use (p<0·001) -GI symtoms (aOR = 11.5, 95%CI = 1.23–107) -Non-adherence (aOR = 19.7, 95%CI = 1.66–234.4) -Baseline AIDS (aOR = 20.2, 95%CI = 1.12–363.6) aOR adjusted odds ratio
Munsiff, Clin Infect Dis 1997 [38]	Cases: HIV-TB co-infected patients with confirmed acquired rifamycin monoresistance n = 29 Control: HIV-TB co-infected patients with drug sensitive TB n = 58	N/A	N/A	N/A	-Non-adherence (OR 11.0, p<0.001) -Baseline AIDS (OR 5.6, p = 0.005])-Baseline smear positivity (OR = 4.1, p = 0.02)
Weiner Clin Infect Dis 2005 [39]	Total in TBTC Study 22 n = 169 Culture confirmed TB, HIV co-infected on intermittent dosing rifabutin regimen who participated in PK sub-study n = 102. Cases of ADR, n = 7. Controls n = 95	Not specified	WC 7/102 (6.9)	Not specified	-Lower baseline CD4 lymphocyte count (p = 0.001) -Rifabutin area under curve (AUC_0-24_, p = 0.01) and maximal concentration (p = 0.03)**This association remained, having adjusted for CD4 lymphocyte count

Abbreviations: WC whole cohort denominator known DST; WCFU denominator f/u DST; PS denominator initial pan-sensitivity; MR denominator initial monoresistance; PR denominator initial polyresistance DOT directly observed therapy IP intensive phase CP continuation phase Hb haemoglobin ART antiretroviral therapy BMI body mass index INH isoniazid PZA pyrazinamide

### Risk factors associated with ADR

Tables 2–5 summarise significant associations and trends for ADR. S4 Table details all covariates that were examined as potential risk factors.

Studies varied considerably in the potential risk factors examined. The disease burden and pathogen factors most frequently examined were baseline mono and polyresistance (16/32), smear positivity (8/32) and cavitatory disease (7/32). Host immune factors most frequently examined were HIV co-infection (10/32) and CD4 lymphocyte count in HIV-infected patients (8/32). The most frequent sociodemographic covariate examined was age (11/32). The most frequently examined programmatic factor was self-administered therapy (SAT) versus directly observed therapy (DOT) (8/32).

### Disease burden and pathogen factors

**Fig 2:**
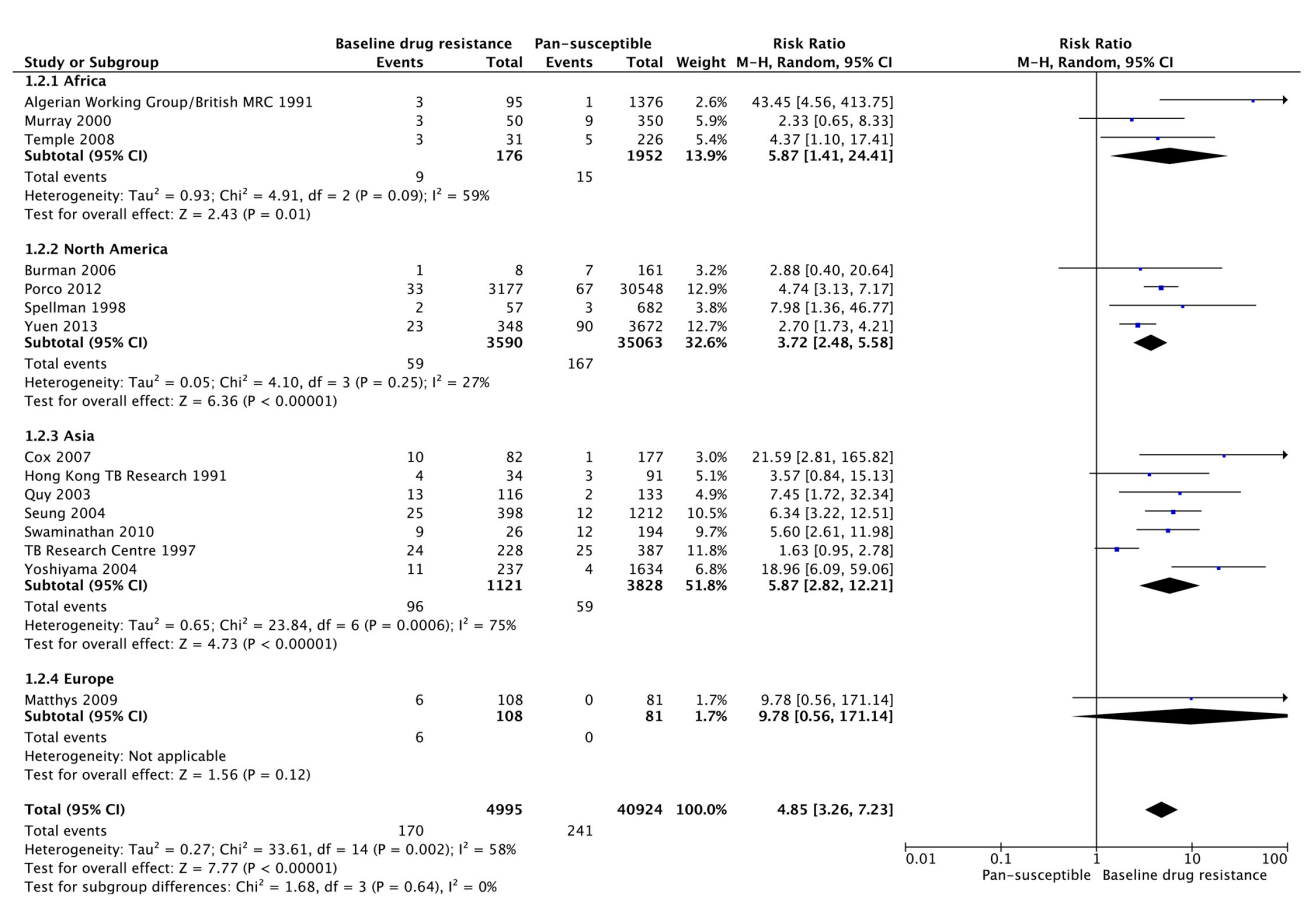
Forest plot of comparison: 1) baseline drug resistance vs pansusceptible MTB, outcome of ADR: 1.2) ADR by region. * 1 study was excluded as we were unable to obtain the exact proportion of patients in the study with non-MDR baseline drug resistance and baseline pan-susceptibility either from the paper or by contacting the authors. The endpoint used for the plot for 12 studies was acquisition of isoniazid/rifamycin/multidrug resistance [3,9,11,13,14,17,19,21,30,31,32,33] and the end point for 3 studies was acquisition of rifamycin resistance [8,28,35], based on data available.

Baseline drug resistance was positively associated with ADR in 15/16 studies that examined its association. In our meta-analysis of 15 studies (including 45,919 patients), baseline drug resistance (monoresistance or polyresistance) was found to be a significant risk factor for ADR (RR 4.85, 95% CI 3.26 to 7.23), when compared with patients with baseline pan-susceptible MTB (Fig 2). There was moderate heterogeneity of the data as evidenced by I^2^ 58% (95% CI 26 to 76%), the same positive trend was seen in all 15 studies included.

**Fig 3:**
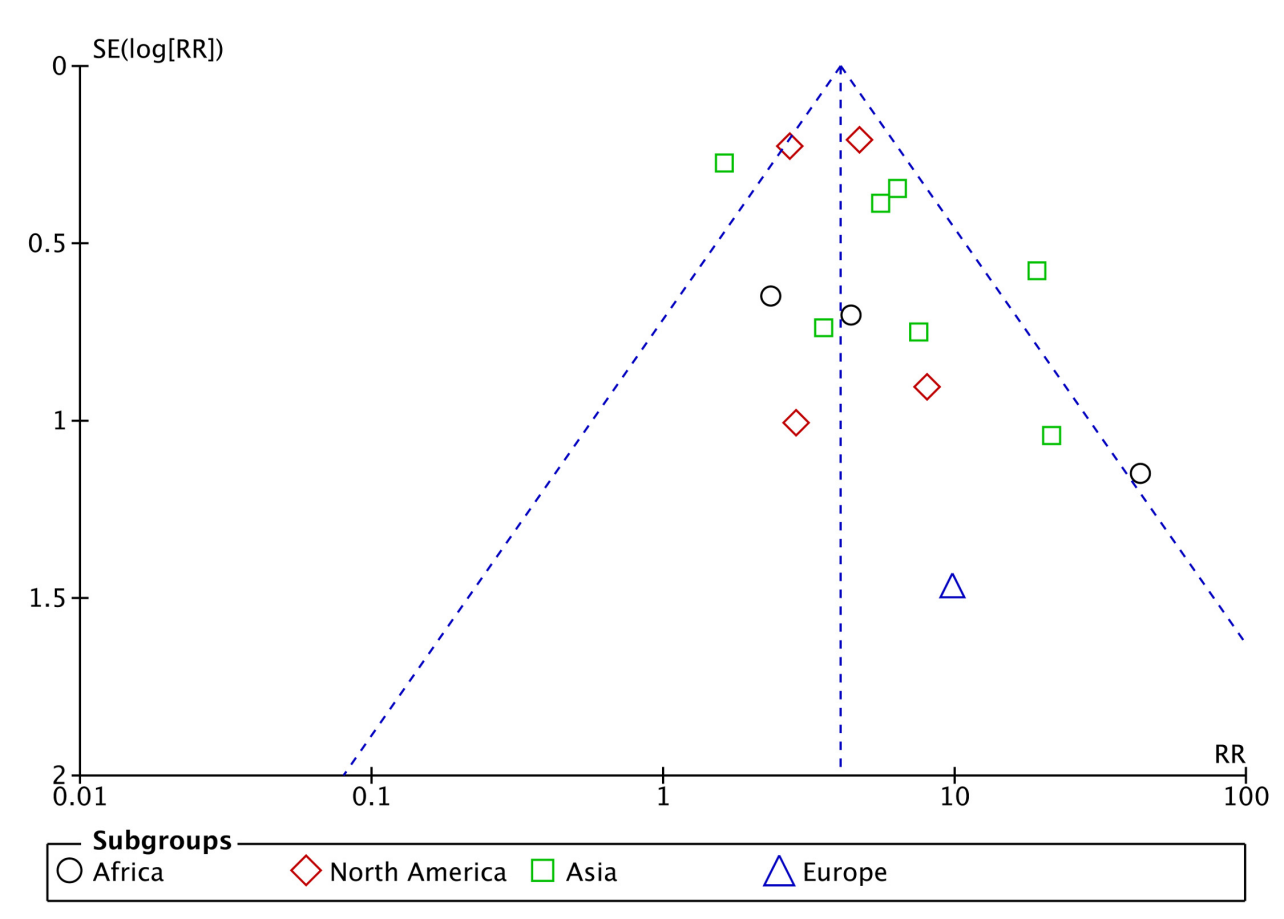
Funnel plot of studies included in meta-analysis of baseline drug resistance and ADR.

A funnel plot for the meta-analysis of baseline drug resistance as a risk factor for ADR (Fig 3) showed a dearth of smaller studies reporting negative effects. However, the asymmetry of the funnel plot also appears to be related to substantial heterogeneity among the larger studies with small standard errors, around the summary estimate of effect, with a resulting imbalance toward a large positive effect estimate.

ADR was significantly associated with extrapulmonary/disseminated TB in 3/5 (60%) studies; with smear positivity in 4/8 (50%) studies; and with extensive radiological disease and cavitatory disease in 1/4 (25%) and 1/7 (14%) studies respectively. *M*. *tuberculosis* complex strain was a risk factor for ADR in 2/4 (50%) studies that examined its role: 1 found increased risk with Beijing strains and 1 with *M*. *bovis*.

### Host immunity and PK variability

**Fig 4:**
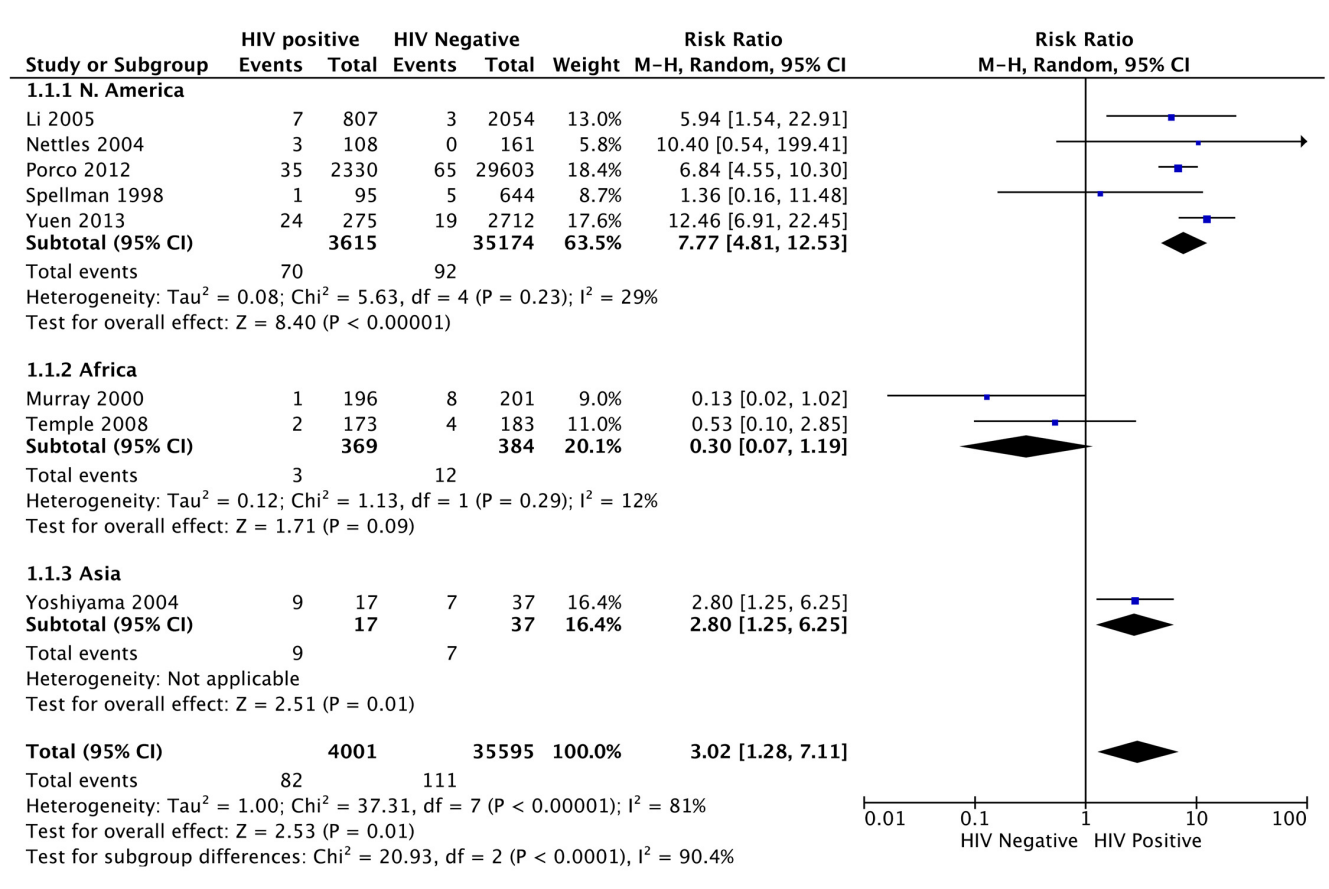
Forest plot of comparison: 1) HIV status, outcome of ADR. 1.2) ADR by region. Only 8/10 studies which examined HIV as a risk factor for ADR were included as we were unable to obtain the exact proportion of those HIV seropositive among the patients that developed ADR from either the paper or by contacting the authors in the other 2 studies. The end point for 5 studies was acquisition of isoniazid/rifamycin/multidrug resistance [13,14,21,30,33] and the end point for 3 studies was acquisition of rifamycin resistance [22,28,35], based on data available.

HIV co-infection was a risk factor for ADR in 8/10 (80%) studies that assessed it. In a meta-analysis of 8/10 studies (35,595 patients), HIV was a significant risk factor for ADR (RR 3.02, 95% CI 1.28 to 7.11) with overall high heterogeneity I^2^ 81% (95% CI 64 to 90%) (Fig 4). Sub-group analysis by continent for ADR showed a RR of 3.23 (95% CI 1.02 to 10.26) with HIV co-infection in 5 North American studies (heterogeneity I^2^ 29%, 95% CI 0 to 72%) whilst there was a trend towards a negative association in 2 African studies (RR 0.3, 95% CI 0.07 to 1.19) with heterogeneity I^2^ 12%.

**Fig 5:**
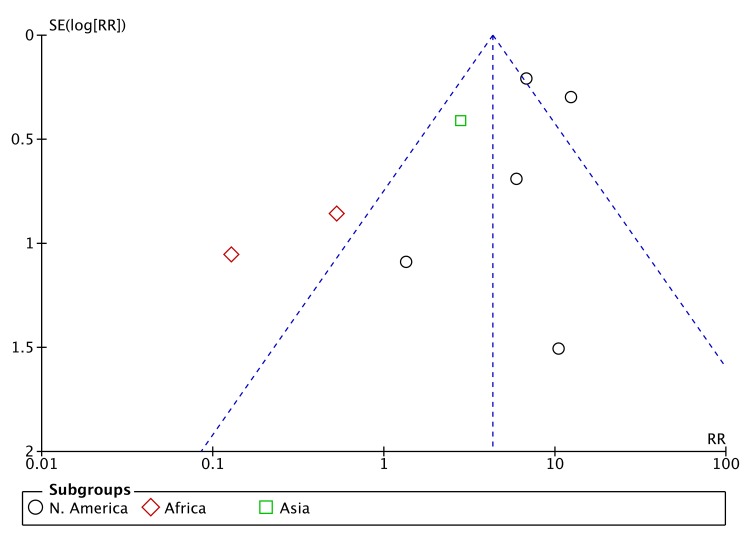
Funnel plot of studies included in meta-analysis of HIV and ADR.

A funnel plot for the meta-analysis of HIV as risk factor of ADR indicated little risk of publication bias (Fig 5).

A low CD4 lymphocyte count at diagnosis in 5/8 studies (63%) and an AIDS diagnosis in 2/2 studies were significant risk factors for ADR amongst HIV-infected patients. Gastrointestinal symptoms at baseline were associated with ADR in 1/1 study and concurrent use of antifungal azoles in 2/2 studies. PK variability was found to be a risk factor for ADR in both studies examining its role. Weiner *et al* found that a lower area under the curve (AUC_0-24hr_) and lower peak concentration (C_max_) for rifabutin was associated with increased risk of ADR. This was in a sub-cohort of patients who were sampled during continuation phase therapy. There was no significant difference in isoniazid C_max_ or AUC_0-24hr_ in cases of ADR, compared with controls. Pasipanodya *et al* found that low rifampicin and isoniazid peak concentrations and AUC_0-24hr_ preceded ADR in 3 patients.

### Sociodemographic factors

Older age [4/11(36%)], foreign birth [1/3 (33%)], ethnicity [2/5 (40%)], unemployment [1/1], substance abuse [2/4 (50%)] and homelessness [1/3 (33%)] were found to be risk factors for ADR in certain studies.

### TB regimen and adherence

Non-adherence was assessed as a risk factor for ADR in 5/32 studies and was associated with ADR in 3/5 (60%) of studies. Directly observed therapy was a risk factor for ADR in 1/8 (12.5%) studies that compared the practice of SAT with DOT. In contrast, SAT was found to be a risk factor for ADR in 4/8 (50%) studies. There was no association between DOT or SAT and ADR in 3 studies. Separate drug formulation, as opposed to fixed dose combination (FDC), was found to be a risk factor for ADR in 1/3 (33%) of studies. Use of rifampicin in the regimen only during intensive phase [16] and lack of ethambutol [18] in a twice/once weekly dosing regimen were associated with cases of ADR in individual RCTs carried out in the 1990s. In one retrospective cohort study, in a sub-analysis of HIV co-infected patients, intermittent dosing of rifampicin during the intensive phase and use of rifampicin instead of rifabutin was associated with ADR [29]. This was in contrast to sub-analysis of HIV-infected patients in another study where there was no significant difference in ADR comparing rifampicin and rifabutin-based regimens [23]. In a RCT, a once weekly rifapentine based regimen in continuation phase was associated with ADR in HIV co-infected individuals [9].

### Cumulative incidence of ADR

Tables 2–5 report DST data and cumulative incidence of acquired isoniazid, rifamycin and MDR for individuals studies stratified by whole cohort, whole cohort with follow up DST, baseline pan-susceptibility, baseline mono-resistance and baseline poly-resistance. In 25 studies, which reported acquired MDR, when considering the overall cohort as denominator, the weighted median incidence of acquired MDR was 0.1% (5^th^ to 95^th^ percentile 0.07 to 3.2%). In 20 studies reporting acquired isoniazid resistance, when considering the overall cohort as denominator, the weighted median incidence of acquired isoniazid resistance was 0.1% (5^th^ to 95^th^ percentile 0.1 to 0.7%). In the 27 studies reporting acquired rifamycin resistance, when considering the overall cohort as denominator, the weighted median incidence of acquired rifamycin resistance was 0.1% (5^th^ to 95^th^ percentile 0.09 to 0.7%). In patients with baseline pan-susceptibility (data available in 15 studies) the weighted median incidence of acquired MDR was 0.2% (5^th^ to 95^th^ percentile 0 to 0.9%). In those with baseline pan-susceptibility, acquired isoniazid resistance (weighted median incidence 0.3%, 5^th^ to 95^th^ percentile 0.06 to 2.7%) did not appear to be more frequent than acquired rifamycin resistance (weighted median incidence 0.3%, 5^th^ to 95^th^ percentile 0 to 0.9%). The weighted median incidence of acquired MDR in patients with baseline monoresistance (data available in 12 studies) was 1% (5^th^ to 95^th^ percentile 0.79 to 10%). The weighted median incidence of acquired MDR in patients with baseline polyresistance (data available in 7 studies) was 10% (5^th^ to 95^th^ percentile 7.1 to 15.5%). It must be noted, that the above estimates of incidence of ADR refer only to studies included in this review and with our search strategy, we excluded studies in which no cases of ADR occurred.

## Discussion

Although acquired MDR was rare overall [weighted median frequency 0.1%], it was more frequent in certain risk groups such as those with baseline mono or polyresistance. A meta-analysis of 15 studies with a moderately heterogeneous data set showed a RR for ADR of 4.96 in patients with baseline drug resistance compared with baseline pan-susceptible profiles. Studies reporting ADR as a treatment outcome varied in geographical location, HIV co-infection, retreatment proportions and treatment regimens administered during intensive and continuation phase as summarised in Table 1 and S2 Table. Weighted pooled analysis of a highly heterogeneous data set showed an increased risk of ADR (RR 3.02) with HIV co-infection. The data presented disaggregated by continent showed a significant association with HIV co-infection in 5 North American studies whilst there was a trend towards a negative association in 2 African studies. This negative association of HIV with ADR in Africa, may partly be explained by a relatively higher proportion of HIV infected patients who develop ADR dying prior to the detection of ADR. Advanced immunosuppression as reflected by a lower baseline CD4 lymphocyte count or AIDS at diagnosis was a risk factor in HIV co-infected patients. Poor adherence and extrapulmonary/disseminated disease were risk factors for ADR in 60% of studies. There was less conclusive evidence regarding the role of PK variability, strain type, DOT versus self-administered therapy, fixed dose combinations and choice of rifamycin as risk factors.

The wide range in reported incidence of ADR may be partially explained by lack of standardization in reporting. For example, where follow up culture and DST results are missing, either the denominator can be altered to reflect this, or the denominator remains as the original cohort number; the assumption being that those with missing DST did not develop ADR. In this review we have presented cumulative incidence of ADR in individual studies, for both the whole cohort and limited to those with follow up DST data.

Baseline mono or polyresistance has previously been recognised as a significant risk factor for ADR. Lew *et al* [40] carried out a meta-analysis looking at the role of initial drug resistance on TB treatment outcomes. Of note, many studies carried out in the 1970s only used rifamycins during a 2 month intensive phase. Lew *et al* found that the cumulative incidence of ADR increased from 0.8% (95% CI 0.5 to 1%) in baseline pan-susceptible cases to 6% (95% CI 4 to 8%) in baseline monoresistant cases and 14% (95% CI 9 to 20%) in baseline polyresistant cases [40]. A review by Menzies *et al* [41] found that in patients with baseline isoniazid monoresistance, a longer duration of rifampicin, use of streptomycin, daily therapy initially, and treatment with a greater number of effective drugs were associated with reduced risk of ADR. Jacobsen *et al* reported 9% progression to MDR TB in a cohort with baseline isoniazid monoresistance who received 12 months of quadruple therapy [42]. In many resource limited settings, Xpert MTB/RIF is used to test for baseline rifampicin resistance and baseline isoniazid resistance will go undetected. During continuation phase, those with isoniazid monoresistance (particularly high level) who are still culture positive, will be effectively receiving rifampicin monotherapy. Hence, there is potential for amplification of drug resistance.

The role of HIV co-infection in the acquisition of TB drug resistance has been a topic of debate. In an immunocompromised host, there is an increased risk of disseminated TB; the latter being an independent risk factor for ADR. Hence, there may be an increased bacterial burden leading to an increased probability of bacteria undergoing spontaneous mutation. It is also hypothesized that less fit drug resistant strains survive longer in the context of poor immunity, allowing for development of compensatory mutations to restore fitness [43–45]. Some MTB strain types are particularly prevalent in immunocompromised hosts [45]. HIV co-infection may cause changes in gut permeability leading to malabsorption of antituberculous drugs [5,46]. As antiretroviral therapy (ART) becomes increasingly available and guidelines advocate early commencement of ART, it remains to be seen if HIV co-infection will continue to be associated with ADR.

*In vitro* work in hollow fibre models has suggested that PK variability and inadequate dosing of TB drugs may be an important risk factor for ADR [47,48]. This is supported by findings from cohort studies [24,39]. However, these results need to be confirmed in studies with robust determination of PK indices and appropriate controls. Pasipanodya *et al* [49] reviewed the role of N-acetyl-transferase type 2 genotype in acquired isoniazid resistance. The link they found between slow acetylator status and ADR may be less significant in the context of currently utilised rifampicin-containing multidrug regimens.

Two studies showed an association with MTB strain. Cox *et al* found an association between Beijing strain and ADR [20]. In a database of 3696 MTB complex strains, 72% of which were Euro-American lineage, only *M*. *bovis* was associated with ADR [36]. Luria-Delbrück fluctuation analyses have suggested that MTB lineage 2 (Beijing) strains are associated with increased mutation rates and acquisition of drug resistance [50]. This may potentially be through sign epistasis where there is favourable interaction between drug resistance mutations and genetic background of the strain [51].

There is no standardized means of measuring adherence and the measure chosen depends on the setting, burden of disease, infrastructure and resources available. Whilst some studies have used DOT (as opposed to SAT), as a surrogate measure of adherence, we have not made this assumption as the outcome of DOT may be confounded by its indication. We have examined non-adherence as a separate risk factor to DOT versus SAT. Non-adherence was a significant risk factor in 3/5 [9,36,37] of the studies that examined its association with ADR. For the 2 studies which showed no significant association between non-adherence and ADR, there was a trend of positive association for 1 of the studies [26] but in the other, all 5 cases of ADR were noted to be adherent with therapy. The impact of DOT versus SAT on ADR was less clear with a protective effect of DOT seen in 50% of studies. A meta-analysis by Pasipanodya *et al* showed no increased risk of microbiologic failure, relapse, or ADR with DOT compared with SAT [52]. Intermittent dosing frequency has been linked with adverse outcomes, including ADR, when administered during intensive phase [8,29], particularly in the context of HIV co-infection.

There are several limitations to this review. The primary focus of the review was evaluating risk factors for ADR. It is not possible to gather any meaningful data regarding risk factors for an event from a study in which no events are reported and consequently, studies that either reported ADR but no risk factors or 0% ADR were excluded and this potentially affected the estimates of ADR cumulative incidence, which was a secondary analysis. There was incomplete MTB strain genotyping to rule out the possibility of dual mixed infection or exogenous re-infection. Only 47% of studies confirmed ADR with identical MTB genotype at baseline and follow-up. Even where genotyping was part of the study design, in some, a proportion of suspected ADR isolates were not available for genotyping [9,14,25,28]. Many studies were retrospective and had small sample sizes and missing DST. Hence, some studies were likely to have been underpowered and there may have been misclassification bias. There were no statistical analyses of risk factors for ADR in 13 studies because the primary outcome was not ADR. We were limited to noting trends of risk for ADR in the studies. We only conducted meta-analyses of HIV co-infection and baseline drug resistance as risk factors. For the meta-analyses undertaken, the weighted estimates of effect size, must be taken in context of moderate to high heterogeneity in the random effects model [53,54]. This heterogeneity is not surprising, considering different geographical populations, varying MTB strains, different regimens and dosing frequencies, different programmatic factors such as self-administered vs DOT and different proportions of retreatment vs new patients. There were also differences in study methodology such as choice of denominator in the calculation of cumulative incidence of ADR and lack of confirmatory genotyping in all studies.

Previous reviews have focussed on a specific risk factor such as fixed dose combination vs. separate drug formulation [55], duration and dosing frequency of rifamycin [56] and baseline isoniazid monoresistance[40]. The strength of this review is that it consolidates the multifactorial aetiology of ADR within a single systematic review.

In conclusion, baseline drug resistance and HIV co-infection were significant risk factors for ADR. Overall, there were limitations of the current evidence and difficulties in evaluating possible contributors to ADR with heterogeneity secondary to both clinical and/or methodological diversity. Although the data are variable, disseminated disease and non-adherence had positive trends of association for ADR. There are likely many other variables contributing to acquired rifamycin and/or isoniazid resistance and studies to date have not adequately evaluated factors such as PK variability and MTB strain type as risk factors for ADR. The multifactorial aetiology ADR in a programmatic setting should be further evaluated via appropriately designed studies.

## Supporting Information

S1 PRISMA Checklist (DOC)

S1 File RevMan data. (RM5)

S1 Table Characteristics of included studies including location and year, criteria for repeat DST and technique used. (DOCX)

S2 Table Aggregate data of studies included in the review. (DOCX)

S3 Table Study quality based on criteria developed in the Critical Appraisal Skills Programme. (DOCX)

S4 Table Risk factors for acquired drug resistance examined. (DOCX)
