# Correction: Risk Factors for Acquired Rifamycin and Isoniazid Resistance: A Systematic Review and Meta-Analysis

**DOI:** 10.1371/journal.pone.0142276

**Published:** 2015-11-18

**Authors:** The PLOS ONE Staff

## Notice of Republication

This article was republished on October 21, 2015, to replace the original Figs 2–5 with Tables 2–5 in order to correct formatting errors, and to remove the tracked changes from S1 and S4 Tables. The article was republished again on November 9, 2015, to correct formatting errors in Table 2; the publisher apologizes for this error. Please download this article again to view the correct version. The originally published, uncorrected article and the republished, corrected article are provided here for reference.

## Supporting Information

S1 File Originally published, uncorrected article. (PDF)

S2 File Republished, corrected article. (PDF)
