# Supplementary material for: Risk Factors for Acquired Rifamycin and Isoniazid Resistance: A Systematic Review and Meta-Analysis
Source: PLoS One. 2015 Sep 25;10(9):e0139017. doi: 10.1371/journal.pone.0139017 (PMC4583446; doi:10.1371/journal.pone.0139017)
Supplement: S1 Table — (DOCX) [file pone.0139017.s003.docx]

**S1 Table: Criteria for repeat DST and technique used**

| Reference | Criteria for repeat DST | DST technique |
| --- | --- | --- |
| Algerian Working Group/British MRC 1991 Tubercle [16] | All positive cultures at end of Rx | LJ proportions method |
| Hong Kong TB Research Centre Madras/BMRC Am Rev Resp Disease 1991 [12] | Cultures performed every month for first 2 years | Not specified |
| Lienhardt JAMA 2011 [17] | Cultures performed at month 2,3,5,6 and during follow up- DST done on positive cultures | LJ proportions method |
| Swaminathan AJRCCM 2010 [10] | If culture positive (tested monthly) | LJ proportions method |
| TB Research Centre IJTLD 1997 [18] | Unfavourable response including culture positive in last 2 months of Rx | MIC phenotypic method |
| Vernon Lancet 1999 [8] | If cultures positive (repeated monthly) | LJ proportions method |
| Aung, IJTLD 2012 [19] *operational study with randomisation | Smear defined failures and relapses | rpoB sequencing |
| Burman AJRCCM 2006 [9] | Relapse or failure | Proportions method & sequencing of rpoB |
| Cox, Clin Infect Dis 2007 [20] | Smear positivity at end of IP or 2 months into CP | LJ proportions method, Bactec 480 |
| El Sahly, J of Infect, 2006 [21] | Not specified | Not specified |
| Murray SAMJ 2000 [22] | Culture +ve at 6 months | Bactec 460 |
| Nettles, Clin Infect Dis 2004 [23] | TB recurrence | Bactec 460 and agar proportions method |
| Pasipanodya , J Inf Dis 2013 [24] | If culture positive at 6, 9, 12, 18 and 24 months | Bactec 460 |
| Temple CID 2008 [14] | If culture positive at 5months | Bactec |
| Chien, JAC 2013 [25] | At 2 and 5 months of treatment | Modified proportional disc elution method |
| Driver, Clin Infect Dis, 2001 [26] | Recurrence or relapse | Not specified |
| Gelmanova, Bull WHO, 2007 [27] | DST at 2,3,5 months and 6 month intervals thereafter | LJ absolute concentration method |
| Jasmer, AJRCCM, 2004 [28] | If culture positive post treatment | Not specified |
| Kim BMC ID 2008 [13] | Not specified | LJ absolute concentrations method |
| Li CID 2005 [29] | If culture +ve after 4 months | Not specified |
| Matthys, PLoS ONE, 2009 [11] | If culture positive at 3 months and 8 months | LJ proportions method |
| Moulding IJTLD 2004 [30] | Positive cultures at 3 month intervals | Bactec (proportion method for problem cultures) |
| Porco CID 2012 [31] | Poor clinical improvement, if culture positive at the end of treatment | Not specified |
| Quy IJTLD 2003 [32] | Smear positivity at 5 or 8 months | LJ proportions method |
| Seung CID 2004 [33] | At 2 and 5 months of treatment | LJ proportions method |
| Spellman 1988 AIDS [34] | Not specified | Not specified |
| Weis, NEJM 1994 [35] | Not specified | Not specified |
| Yoshiyama IJTLD 2004 [15] | If culture positive at the end of treatment | LJ proportions method |
| Yuen, PLoSONE 2013 [36] | Not specified | Not specified |
| Bradford Lancet 1996 [37] | Relapse or failure | Proportions method or Bactec 460 |
| Munsiff, Clin Infect Dis 1997 [38] | Not specified | Agar proportions method or Bactec |
| Weiner CID 2005 [39] | Treatment failure or relapse | Proportions method |

**LJ Lowenstein-Jensen MGIT mycobacterial indicator growth tubes**
