# Supplementary material for: Risk Factors for Acquired Rifamycin and Isoniazid Resistance: A Systematic Review and Meta-Analysis
Source: PLoS One. 2015 Sep 25;10(9):e0139017. doi: 10.1371/journal.pone.0139017 (PMC4583446; doi:10.1371/journal.pone.0139017)
Supplement: S2 Table — (DOCX) [file pone.0139017.s004.docx]

**S2 Table. Aggregate data of studies included in the review**

| Characteristics of studies (N=32) | Number of studies (%) |
| --- | --- |
| Year study completed |  |
| 1980- 2000 | 15 (47) |
| After 2000 | 17 (53) |
| Geographical location* |  |
| North America | 15(47) |
| South America | 1 (3) |
| North Africa | 2 (6) |
| South and East Africa | 4 (12.5) |
| Indian subcontinent | 4 (12.5) |
| South East Asia | 5 (16) |
| Former Soviet Union states | 4 (12.5) |
| HIV co-infection |  |
| HIV co-infection data reported for cohort | 25 (78) |
| All patients in cohort had HIV | 5 (16) |
| Presumed 0% HIV co-infection in cohort | 4 (12.5) |
| Retreatment |  |
| Proportion patients receiving retreatment for TB reported for cohort | 19 (59) |
| Retreatment patients excluded | 6 (19) |
| Criteria for repeat DST |  |
| Unfavourable treatment response such as smear or culture positivity at the end of treatment or clinical relapse | 15 (47) |
| Cultures were done at regular intervals throughout study and follow up and DST carried out for positive culture | 10 (31) |
| Not specified | 7 (22) |
| Genotype carried out to confirm ADR | 15 (47) |

DST drug sensitivity testing ADR acquired rug resistance *some studies were carried out in more than 1 continent
