# Supplementary material for: Risk Factors for Acquired Rifamycin and Isoniazid Resistance: A Systematic Review and Meta-Analysis
Source: PLoS One. 2015 Sep 25;10(9):e0139017. doi: 10.1371/journal.pone.0139017 (PMC4583446; doi:10.1371/journal.pone.0139017)
Supplement: S3 Table — (DOCX) [file pone.0139017.s005.docx]

**S3 Table. Study quality based on criteria developed in the Critical Appraisal Skills Programme**

| Study ID | Study design | Was LTFU significant? | Was study appropriately powered? | Was blinding appropriate? | Was randomisation appropriate? | Apart from intervention were groups treated equally? | Was treatment effect measured precisely? | Was study population representative | Was exposure accurately measured to minimise bias? | Have confounders been accounted for? | Were cases and controls defined precisely? | Were controls appropriate? |
| --- | --- | --- | --- | --- | --- | --- | --- | --- | --- | --- | --- | --- |
| Algerian Working Group/British MRC 1991 Tubercle [16] | RCT | No | Yes | N/A | N/A | Yes | Yes | N/A | N/A | N/A | N/A | N/A |
| Hong Kong TB Research Centre Madras/BMRC Am Rev Resp Disease 1991 [12] | RCT | Yes | No | Yes | Yes | Yes | Yes | N/A | N/A | N/A | N/A | N/A |
| Lienhardt JAMA 2011 [17] | RCT | No | Yes | N/A | Yes | Yes | Yes | N/A | N/A | N/A | N/A | N/A |
| Swaminathan AJRCCM 2010 [10] | RCT | No | Yes | N/A | Yes | Yes | Yes | N/A | N/A | N/A | N/A | N/A |
| TB Research Centre IJTLD 1997 [18] | RCT | No | Yes | N/A | UC | No | Yes | N/A | N/A | N/A | N/A | N/A |
| Vernon Lancet 1999 [8] | RCT | No | No | N/A | Yes | Yes | Yes | N/A | N/A | N/A | N/A | N/A |
| Aung, IJTLD 2012 [19] *operational study with randomisation | Cohort | No | Yes | NA | NA | NA | NA | Yes | No | No | N/A | N/A |
| Burman AJRCCM 2006 [9] | Cohort | No | Yes | NA | NA | NA | NA | No | Yes | Yes | N/A | N/A |
| Cox, Clin Infect Dis 2007 [20] | Cohort | No | Yes | NA | NA | NA | NA | Yes | Yes | No | N/A | N/A |
| El Sahly, J of Infect, 2006 [21] | Cohort | No | Yes | NA | NA | NA | NA | Yes | Yes | Yes | N/A | N/A |
| Murray SAMJ 2000 [22] | Cohort | No | No | NA | NA | NA | NA | No | Yes | No | N/A | N/A |
| Nettles, Clin Infect Dis 2004 [23] | Cohort | No | Yes | NA | NA | NA | NA | Yes | Yes | Yes | N/A | N/A |
| Pasipanodya , J Inf Dis 2013 [24] | Cohort | No | Yes | NA | NA | NA | NA | No | Yes | Yes | N/A | N/A |
| Temple CID 2008 [14] | Cohort | No | Yes | NA | NA | NA | NA | No | Yes | Yes | N/A | N/A |
| Chien, JAC 2013 [25] | Cohort | No | Yes | NA | NA | NA | NA | Yes | Yes | Yes | N/A | N/A |
| Driver, Clin Infect Dis, 2001 [26] | Cohort | Not stated | Yes | NA | NA | NA | NA | Yes | Yes | Yes | N/A | N/A |
| Gelmanova, Bull WHO, 2007 [27] | Cohort | No | Yes | NA | NA | NA | NA | Yes | Yes | Yes | N/A | N/A |
| Jasmer, AJRCCM, 2004 [28] | Cohort | No | Yes | NA | NA | NA | NA | Yes | Yes | Yes | N/A | N/A |
| Kim BMC ID 2008 [13] | Cohort | No | No | NA | NA | NA | NA | No | Yes | Yes | N/A | N/A |
| Li CID 2005 [29] | Cohort | No | Yes | NA | NA | NA | NA | Yes | Yes | Yes | N/A | N/A |
| Matthys, PLoS ONE, 2009 [11] | Cohort | No | No | NA | NA | NA | NA | No | Yes | Yes | N/A | N/A |
| Moulding IJTLD 2004 [30] | Cohort | No | Yes | NA | NA | NA | NA | No | No | No | N/A | N/A |
| Porco CID 2012 [31] | Cohort | No | Yes | NA | NA | NA | NA | Yes | Yes | Yes | N/A | N/A |
| Quy IJTLD 2003 [32] | Cohort | No | Yes | NA | NA | NA | NA | Yes | Yes | No | N/A | N/A |
| Seung CID 2004 [33] | Cohort | No | Yes | NA | NA | NA | NA | Yes | Yes | No | N/A | N/A |
| Spellman 1988 AIDS [34] | Cohort | No | Yes | NA | NA | NA | NA | Yes | Yes | Yes | N/A | N/A |
| Weis, NEJM 1994 [35] | Cohort |  |  | NA | NA | NA | NA |  |  |  | N/A | N/A |
| Yoshiyama IJTLD 2004 [15] | Cohort | No | No | NA | NA | NA | NA | No | Yes | No | N/A | N/A |
| Yuen, PLoSONE 2013 [36] | Cohort | No | Yes | NA | NA | NA | NA | Yes | Yes | Yes | N/A | N/A |
| Bradford Lancet 1996 [37] | Case-control | N/A | N/A | N/A | N/A | N/A | N/A | N/A | N/A | Yes | Yes | Yes |
| Munsiff, Clin Infect Dis 1997 [38] | Case-control | N/A | N/A | N/A | N/A | N/A | N/A | N/A | N/A | Yes | Yes | Yes |
| Weiner CID 2005 [39] | Case-control | N/A | N/A | N/A | N/A | N/A | N/A | N/A | N/A | Yes | Yes | Yes |
