# Supplementary material for: Risk Factors for Acquired Rifamycin and Isoniazid Resistance: A Systematic Review and Meta-Analysis
Source: PLoS One. 2015 Sep 25;10(9):e0139017. doi: 10.1371/journal.pone.0139017 (PMC4583446; doi:10.1371/journal.pone.0139017)
Supplement: S4 Table — (DOCX) [file pone.0139017.s006.docx]

S4 Table. Risk factors for acquired drug resistance examined

| Randomised controlled trials | |
| --- | --- |
| Reference | Potential risk factors for acquired drug resistance analysed |
| Algerian Working Group/British MRC 1991 Tubercle [16] | Rifampicin in regimen only during intensive phase * |
|  | Baseline resistance to INH and STREP * |
| Hong Kong TB Research Centre Madras/BMRC  Am Rev Resp Disease 1991 [12] | 6 vs 8 months treatment including RHSZ throughout * |
|  | Baseline drug resistance * |
| Lienhardt JAMA 2011 [17] | Fixed dose combination in IP vs separate drug formulation * |
| Swaminathan AJRCCM 2010 [10] | Baseline median CD4 count |
|  | Baseline median HIV viral load |
|  | Nonadherence |
|  | Baseline isoniazid resistance |
|  | 4 vs 7 month continuation phase |
|  | Baseline weight |
|  | Baseline Haemoglobin |
|  | Sputum smear grade |
| TB Research Centre IJTLD 1997 [18] | Ethambutol in a twice weekly regimen * |
|  | Frequency of dosing (twiceweekly or onceweekly) * |
|  | Baseline drug resistance * |
| Vernon Lancet 1999 [8] | Onceweekly isoniazid/rifapentine |
|  | Baseline CD4 |
|  | Age  Extrapulmonary + pulmonary disease |
|  | Use of antifungal azoles |
| Prospective cohort studies | |
| Reference | Potential risk factors for acquired drug resistance analysed |
| Aung, IJTLD 2012 [19] # operational study with randomisation | Extension of intensive phase of treatment by 1 month for patients who are smearpositive after 2 months * |
| Burman AJRCCM 2006 [9] | Age |
|  | Sex |
|  | Ethnicity |
|  | Foreign birth (immigrant) |
|  | Baseline BMI |
|  | Baseline resistance to INH/PZA |
|  | Extrapulmonary TB |
|  | Cavitatory disease |
|  | Extensive (bilateral) radiological disease |
|  | Receiving of both rifampin and rifabutin during intensive phase |
|  | Culture positivity at 2months |
|  | Concurrent drugs which reduce rifabutin levels |
|  | Concurrent drugs which increase rifabutin levels |
|  | Baseline HIV viral load |
|  | Lack of use of ART during TB treatment |
|  | Lack of use of ART in first 2 months of TB treatment |
|  | Baseline CD4 lymphocyte count |
| Cox, Clin Infect Dis 2007 [20] | Baseline drug resistance (polyresistance) |
|  | Strain type |
| El Sahly, J of Infect, 2006 [21] | HIV coinfection |
|  | Ethnicity |
|  | Smear positive |
|  | Disseminated TB (with pleural effusion) |
| Murray SAMJ 2000 [22] | Baseline drug resistance * |
|  | HIV coinfection * |
| Nettles, Clin Infect Dis 2004 [23] | HIV coinfection |
|  | Baseline median CD4 lymphocyte count |
|  | Type of rifamycin used (rifampicin vs rifabutin) |
| Pasipanodya , J Inf Dis 2013 [24] | PK variability including peak and 24 hr area under the concentration time curve for drugs R,H,PZA *statistical analyses only performed for a composite outcome of death, treatment failure and relapse, not for acquired drug resistance |
| Temple CID 2008 [14] | Baseline resistance |
|  | Age |
|  | Sex |
|  | Baseline BMI |
|  | Nonadherence |
|  | HIV coinfection |
|  | Baseline CD4 |
|  | ART use |
|  | Baseline extensive radiological disease |
|  | Baseline cavitatory disease |
| Retrospective cohort studies | |
| Reference | Potential risk factors for acquired drug resistance analysed |
| Chien, JAC 2013 [25] | Age group 45-64 |
|  |  |
|  | Smear positivity |
|  | Self-administration of treatment/lack of DOT |
|  | Cavitatory disease |
| Driver, Clin Infect Dis, 2001 [26] | HIV coinfection * |
|  | Selfadministration of treatment/lack of DOT * |
| Gelmanova, Bull WHO, 2007 [27] | Age |
|  | Gender |
|  | Non-adherence |
|  | Side effects |
|  | Substance abuse |
|  | Cavitatory disease |
|  | Previously incarcerated |
|  | Smear positivity |
|  | Treatment commenced in hospital setting |
|  | Hospitalisation later in treatment |
|  | Self-administration of treatment/lack of DOT |
| Jasmer, AJRCCM, 2004 [28] | Self-administration of treatment/lack of DOT |
| Kim BMC ID 2008 [13] | Age * |
|  | Sex * |
|  | Comorbidity * |
|  | Previous TB treatment * |
|  | Length of RE in continuation phase * |
|  | Extensive radiological disease * |
|  | Smear positivity * |
| Li CID 2005 [29] | HIV coinfection |
|  | CD4 count in the HIV coinfected cohort |
|  | Baseline drug resistance in the HIV cinfected cohort |
|  | Change in type of rifamycin (rifampin to rifabutin or vice versa) during treatment |
|  | Intermittent dosing of rifampin or rifabutin during intensive phase |
| Matthys, PLoS ONE, 2009 [11] | Baseline drug resistance * |
| Moulding IJTLD 2004 [30] | Separate drug formulation (as opposed to fixed dose combination) * |
|  | Private sector management * |
| Porco CID 2012 [31] | Age |
|  | Ethnicity |
|  | Gender |
|  | Foreign birth |
|  | Previous TB |
|  | Extrapulmonary TB |
|  | Smear positivity |
|  | Private sector care |
|  | Baseline drug resistance |
|  | HIV coinfection |
|  | Cavitatory disease |
| Quy IJTLD 2003 [32] | Age |
|  | Sex |
|  | Baseline drug resistance |
| Seung CID 2004 [33] | Baseline drug resistance * |
| Spellman 1988 AIDS [34] | HIV coinfection * |
|  | Baseline drug resistance * |
| Weis, NEJM 1994 [35] | Selfadministration of treatment (lack of DOT) |
| Yoshiyama IJTLD 2004 [15] | Previous treatment failure * |
|  | Baseline resistance * |
|  | HIV coinfection * |
|  | Strain type * |
| Yuen, PLoSONE 2013 [36] | Age |
|  | Ethnicity |
|  | Country of birth |
|  | Region of birth |
|  | In correctional facility at time of diagnosis |
|  | Injecting drug use |
|  | Noninjecting drug use |
|  | Alcohol |
|  | Strain type |
|  | HIV coinfection |
|  | Homelessness |
|  | Baseline drug resistance |
|  | Smear positivity |
|  | Cavitatory disease |
|  | Extrapulmonary only disease |
|  | Self-administration of treatment/lack of DOT |
| Case control studies | |
| Reference | Potential risk factors for acquired drug resistance analysed |
| Bradford Lancet 1996 [37] | Ethnicity |
|  | Foreign birth |
|  | Unemployment |
|  | Homelessness |
|  | Alcohol excess |
|  | Baseline AIDS |
|  | Self-administration of treatment/lack of DOT |
|  | ART use |
|  | Previous TB treatment |
|  | Initial 4 drug regimen |
|  | GI symptoms |
|  | Azole use |
|  | Non-adherence |
|  | CD4 lymphocyte count |
|  | Fixed dose RH tablet |
| Munsiff, Clin Infect Dis 1997 [38] | Age |
|  | Gender |
|  | Country of birth |
|  | Recreational drugs |
|  | Homelessness |
|  | Self-administration of treatment/lack of DOT |
|  | Non-adherence |
|  | Baseline AIDS |
|  | Baseline smear positivity |
| Weiner CID 2005 [39] | Age |
|  | Gender |
|  | Ethnicity |
|  | Baseline BMI |
|  | Site of TB involvement |
|  | Extensive (bilateral) radiological disease |
|  | Cavitatory disease |
|  | Rifabutin area under curve (AUC_024hrs_) and maximal concentration |
|  | Deacetylrifabutin under curve (AUC_024hrs_) and maximal concentration |
|  | Isoniazid AUC and maximal concentration |
|  | Birth in Mexico |
|  | Baseline CD4 lymphocyte count |
|  | Baseline HIV viral load |
|  | Deacetylrifabutin AUC and maximal concentration |
|  | Nacetyltransferase type 2 genotype |
| Abbreviations: DOT directly observed therapy NTM nontuberculous mycobacteria MDR multidrug resistant ART antiretroviral therapy BMI body mass index INH/H isoniazid PZA pyrazinamide R rifampin E thambutol IP intensive phase CP continuation phase Hb haemoglobin ART antiretroviral therapy BMI body mass index . * No statistical analysis was performed in the study and therefore the risk factor for ADR was reported as per trend noted | |
