# Supplementary material for: Correction: Risk Factors for Acquired Rifamycin and Isoniazid Resistance: A Systematic Review and Meta-Analysis
Source: PLoS One. 2015 Nov 18;10(11):e0142276. doi: 10.1371/journal.pone.0142276 (PMC4651325; doi:10.1371/journal.pone.0142276)
Supplement: S1 File — (PDF) [file pone.0142276.s001.pdf]

PLOS ONE

RESEARCH ARTICLE

Risk Factors for Acquired Rifamycin and Isoniazid Resistance: A Systematic Review and Meta-Analysis

Neesha Rockwood[1,2]*, **Leila H. Abdullahi**[5], **Robert J. Wilkinson**[1,2,3,4], **Graeme Meintjes**[1,2,4]

1 Department of Medicine, Imperial College, London W2 1PG, United Kingdom, **2** Clinical Infectious Diseases Research Initiative, Institute of Infectious Disease and Molecular Medicine, University of Cape Town, Cape Town, South Africa, **3** Francis Crick Institute Mill Hill Laboratory, London, United Kingdom, **4** Department of Medicine, University of Cape Town, Cape Town, South Africa, **5** Vaccines for Africa Initiative, Institute of Infectious Disease and Molecular Medicine, University of Cape Town, Cape Town, South Africa

* neesha.rockwood@doctors.org.uk

CrossMark
click for updates

OPEN ACCESS

Citation: Rockwood N, Abdullahi LH, Wilkinson RJ, Meintjes G (2015) Risk Factors for Acquired Rifamycin and Isoniazid Resistance: A Systematic Review and Meta-Analysis. PLoS ONE 10(9): e0139017. doi:10.1371/journal.pone.0139017

Editor: Dipankar Chatterji, Indian Institute of Science, INDIA

Received: May 18, 2015

Accepted: September 7, 2015

Published: September 25, 2015

Copyright: © 2015 Rockwood et al. This is an open access article distributed under the terms of the Creative Commons Attribution License, which permits unrestricted use, distribution, and reproduction in any medium, provided the original author and source are credited.

Data Availability Statement: All relevant data are within the paper and its Supporting Information files.

Funding: Robert J. Wilkinson is supported by the Wellcome Trust www.wellcome.ac.uk [WT 104803 ; WT084323], UK MRC www.mrc.ac.uk [UKMRC U1175.02.002.00014 and MRC-SHIP] and the European Union Grants [EU FP7 HEALTH-F3-2012-305578; FP7-PEOPLE-2011-IRSES], UK MRC [UKMRC U1175.02.002.00014] and the European Union ec.europa.eu [EU FP7 HEALTH-F3-2012-305578; FP7-PEOPLE-2011-IRSES]. Graeme Meintjes was supported by a Wellcome Trust fellowship www.wellcome.ac.uk [WT 098316] and in

Abstract

Background

Studies looking at acquired drug resistance (ADR) are diverse with respect to geographical distribution, HIV co-infection rates, retreatment status and programmatic factors such as regimens administered and directly observed therapy. Our objective was to examine and consolidate evidence from clinical studies of the multifactorial aetiology of acquired rifamycin and/or isoniazid resistance within the scope of a single systematic review. This is important to inform policy and identify key areas for further studies.

Methods

Case-control and cohort studies and randomised controlled trials that reported ADR as an outcome during antitubercular treatment regimens including a rifamycin and examined the association of at least 1 risk factor were included. Post hoc, we carried out random effects Mantel-Haenszel weighted meta-analyses of the impact of 2 key risk factors 1) HIV and 2) baseline drug resistance on the binary outcome of ADR. Heterogeneity was assessed used I^2 statistic. As a secondary outcome, we calculated median cumulative incidence of ADR, weighted by the sample size of the studies.

Results

Meta-analysis of 15 studies showed increased risk of ADR with baseline mono- or polyresistance (RR 4.85 95% CI 3.26 to 7.23, heterogeneity I^2 58%, 95% CI 26 to 76%). Meta-analysis of 8 studies showed that HIV co-infection was associated with increased risk of ADR (RR 3.02, 95% CI 1.28 to 7.11); there was considerable heterogeneity amongst these studies (I^2 81%, 95% CI 64 to 90%). Non-adherence, extrapulmonary/disseminated disease and advanced immunosuppression in HIV co-infection were other risk factors noted. The weighted median cumulative incidence of acquired multi drug resistance calculated in 24

part by the National Research Foundation (NRF) www.nrf.ac.za of South Africa [UID: 85858]. The funders had no role in study design, data collection and analysis, decision to publish, or preparation of the manuscript.

Competing Interests: The authors have declared that no competing interests exist.

studies (assuming whole cohort as denominator, regardless of follow up DST) was 0.1% (5th to 95th percentile 0.07 to 3.2%).

Conclusion

Baseline drug resistance and HIV co-infection were significant risk factors for ADR. There was a trend of positive association with non-adherence which is likely to contribute to the outcome of ADR. The multifactorial aetiology of ADR in a programmatic setting should be further evaluated via appropriately designed studies.

Introduction

Resistance to both first line antitubercular drugs rifampicin (of the rifamycin drug class) and isoniazid (multi drug resistant tuberculosis (MDR TB)) is an increasing global health problem. The World Health Organisation (WHO) estimates there were 450,000 cases of MDR TB with 170,000 deaths in 2012 [1]. Cure and completion rates are lower than for drug susceptible TB, with higher mortality rates [2] and there is huge cost to health systems. Whilst transmitted drug resistance has been highlighted as important in fuelling the spread of the epidemic, a better understanding of what factors contribute to the initial emergence of resistance is needed to inform policy. Acquired drug resistance (ADR) is the development, fixation and amplification of mutations conferring resistance under drug pressure during treatment. Verification of true ADR requires ruling out initial dual mixed infection or subsequent exogenous re-infection with a drug resistant strain of *M*. *tuberculosis* (MTB).

ADR has been recognised since chemotherapy was first discovered. The early emergence of ADR with streptomycin monotherapy, heralded the need for multidrug regimens to achieve cure and prevent further accumulation of resistance. The inclusion of rifampicin and pyrazinamide in TB regimens since the 1970s led to shortening of TB regimens from 2 years to 6 months. The rate of stochastic acquired drug resistance has been calculated to be in the order of 2.25×10^{10} mutations per bacterium per generation for rifampicin and 2.56×10^{8} mutations per bacterium per generation for isoniazid [3] within the human host. Upon the background of this natural evolution of resistance, programmatic factors such as problems in maintaining drug supplies and ensuring patient adherence and treatment completion have remained and contributed to the global MDR epidemic through creating the selective pressure necessary for ADR to emerge.

A recently published study of ADR in a hollow fibre model system has questioned the conventional notion that poor adherence accounts for the majority of ADR [4]. Several plausible explanations as to how HIV could predispose to ADR have been proposed including malabsorption of antitubercular drugs [5] and host immunosuppression leading to tolerance of strain-specific polymorphisms in the pathway to drug resistance [6]. However, whether HIV is indeed a risk factor for ADR remains to be clarified. The objective of this review was to consolidate evidence from studies that examined any risk factors for acquired rifamycin and/or isoniazid resistance in patients undergoing antitubercular therapy containing a rifamycin at least during the intensive phase. After conducting the systematic review, a post-hoc decision was taken to carry out 2 separate meta-analyses focused on: 1) HIV infection 2) baseline drug resistance as risk factors for the binary outcome of ADR.

Methods

We followed the Preferred Reporting Items for Systematic Reviews and Meta-Analyses (PRISMA) guidelines. We registered the review in PROSPERO (crd.york.ac.uk CRD42014003856).

Selection criteria

We included case-control and cohort studies and randomised controlled trials (RCTs) reporting ADR as either a primary or secondary adverse outcome. To be included, studies had to examine the association of at least 1 risk factor with ADR. Also, patients of any age needed to be on regimens of at least 6 months' duration which contained rifamycin at least in the intensive phase. We excluded studies that defined ADR as cases of baseline resistance in patients undergoing retreatment for TB. Studies that reported no cases of ADR were excluded. We did not limit our case definition of ADR to studies that had ruled out exogenous re-infection or initial dual mixed infection with different strains using genotypic methods. However, where the data was available, we excluded cases identified as exogenous re-infection via genotyping. Although we collected data on baseline drug sensitivities, the performance of drug sensitivity testing (DST) at baseline in the entire cohort was not required for inclusion. This allowed for inclusion of studies from settings where baseline DST was not routinely performed, but our analyses focussed on those patients in the cohort who did have baseline DST. ADR was defined as identification of new resistance (compared with a baseline isolate of known DST) to rifamycin and/or isoniazid which was made after minimum of 2 weeks on TB treatment or after completion of TB treatment.

Search strategy

Searches were run in Pubmed/MEDLINE, EMBASE, Cochrane Library, Web of Science, Biosis previews and the Trip Database from 1950 to January 2014. In Pubmed, filters were used to select the following languages: Chinese; English; Italian; Russian; Spanish; French. Our keywords were 'tuberculosis' or '*Mycobacterium tuberculosis*' AND 'acquired drug resistance' OR 'amplified drug resistance.' We hand-searched reference lists of reviews and eligible papers for other relevant articles in English.

Study selection, data extraction and quality assessment

Two reviewers (NR, GM) independently assessed the titles and abstracts of studies from the searches based on pre-specified eligibility criteria. If it was unclear from the abstract whether inclusion criteria were met, the full article was reviewed. Any uncertainty or disagreement about eligibility was resolved through discussion.

The two reviewers then independently extracted data using a structured data extraction form. Any disagreements were discussed. In cases of missing or incomplete information authors were contacted. Critical appraisal tools, developed in the Critical Appraisal Skills Programme (CASP) for judging methodological quality of RCTs, cohort and case control studies, were amalgamated and used to judge methodological quality [7].

Data synthesis

Risk factors for ADR were tabulated for all studies. If univariate or multivariate analyses were performed, then only if there was a significant association with ADR was the factor categorised as 'risk factor for ADR'. If no statistical analysis was performed but a risk factor for ADR was described in the study, it was reported as per trend noted. Random effects meta-analyses with

Mantel-Haenszel weighting were performed for the covariates baseline drug resistance and HIV co-infection for the binary outcome of ADR using the Cochrane Collaboration Review Manager Version 5.3 statistical software. We calculated risk ratios (RR) and their corresponding 95% confidence intervals (CI) and p-values. Heterogeneity between studies was assessed by calculating the I^2 statistic and its corresponding 95% CI using Stata version 13.1. As a secondary outcome, the cumulative incidence of acquired isoniazid, rifamycin and MDR was reported for individual studies. Patients with known baseline MDR were excluded from calculations. When feasible, the incidence of ADR was calculated using the following denominators: 1) as a proportion of the whole cohort, 2) as a proportion of those with follow up DST, 3) as a proportion of those with baseline pan-susceptibility, 4) as a proportion of those with baseline monoresistance and 5) as a proportion of those with baseline polyresistance. The median cumulative acquired isoniazid, acquired rifamycin and acquired MDR incidence across all studies that reported these was also calculated, weighted by the overall sample size of each study.

Results

Study selection and assessment

We identified 798 citations through the electronic database searches: 703 were excluded after abstract review. Another 26 studies were identified through reference review. One hundred full text articles were examined and 32 deemed eligible (6 RCTs, 8 prospective cohort, 15 retrospective cohort and 3 case control studies) (Fig 1).

Table 1, S1 and S2 Tables provide detailed break down and aggregate data of studies included in the review. Certain studies restricted inclusion to specific populations: those with HIV co-infection (n = 5) [8–10], those incarcerated (n = 1) [11], those with silicotuberculosis (n = 1) [12], those with isoniazid monoresistance (n = 1) [13] and retreatment patients (n = 2) [14–15]. S3 Table provides an appraisal of study quality [7]. Loss to follow up was not noted to be significant (pre-defined threshold 20%) in any study. We assessed that in all selected RCTs, treatment effect was measured precisely. We assessed in 20/22 cohort studies, exposure was accurately measured to minimise bias. As illustrated in Figs 2–5, only a proportion of individuals included as the 'whole cohort' at baseline had follow up DST as per criteria detailed in S1 Table. These criteria ranged from being performed at a regular monthly interval in all culture positive isolates; to those who were smear/culture positive at 2 and 5–6 months; to being only performed in cases of suspected failure/relapse. In some cases, this may have compromised accuracy of measurement of outcome.

Risk factors associated with ADR

Figs 2–5 summarise significant associations and trends for ADR. S4 Table details all covariates that were examined as potential risk factors.

Studies varied considerably in the potential risk factors examined. The disease burden and pathogen factors most frequently examined were baseline mono and polyresistance (16/32), smear positivity (8/32) and cavitatory disease (7/32). Host immune factors most frequently examined were HIV co-infection (10/32) and CD4 lymphocyte count in HIV-infected patients (8/32). The most frequent sociodemographic covariate examined was age (11/32). The most frequently examined programmatic factor was self-administered therapy (SAT) versus directly observed therapy (DOT) (8/32).

Disease burden and pathogen factors. Baseline drug resistance was positively associated with ADR in 15/16 studies that examined its association. In our meta-analysis of 15 studies (including 45,919 patients), baseline drug resistance (monoresistance or polyresistance) was found to be a significant risk factor for ADR (RR 4.85, 95% CI 3.26 to 7.23), when compared

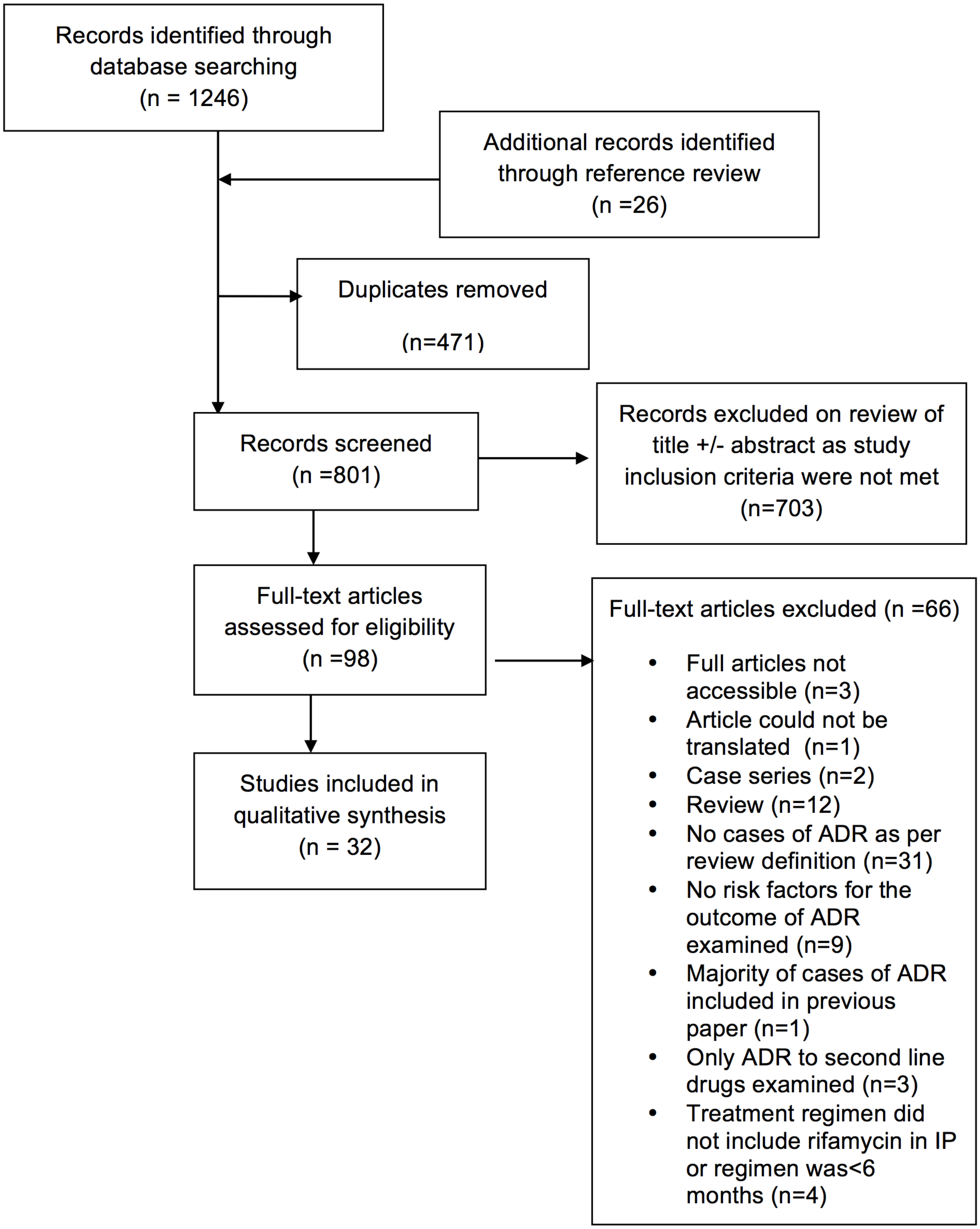

Fig 1. Summary of literature search and study selection.

doi:10.1371/journal.pone.0139017.g001

Table 1. Characteristics of included studies including HIV co-infection, proportion receiving retreatment, treatment regimen, whether treatment was self-administered or directly observed and use of genotyping.

Reference	Study location and year	HIV prevalence	Retreatment (ReRx)	Regimen	DOT	Genotyping carried out in a proportion of available isolates
RCTs						
Algerian Working Group/British MRC 1991 Tubercle [16]	Algeria Oct 1981-Dec1983	0%	Not specified	IP: Regimen 1) 2($HRSZ_7$) Regimen 2) 2($HREZ_7$) CP: Regimen 1) 4(HR_7) Regimen 2) 2(HR_7)4(H_7)	DOT in IP (whilst on streptomycin)	No
Hong Kong TB Research Centre Madras/BMRC Am Rev Resp Disease 1991 [12]	Hong Kong Dec 1980- Dec 1985	Not specified	Not specified	Regimen 1) 6(RHSZ) Regimen 2) 8($RHSZ)_3$ (E was added for first 3 months if retreatment patient)	100%	No
Lienhardt JAMA 2011 [17]	Algeria, Colombia, Guinea, Vietnam, Peru,Mozambique, Tanzania, Bolivia 2003–2008	6.6%	0%	IP: Regimen 1) 2($RHEZ_7$) as FDC Regimen 2) 2 ($RHEZ_7$) as single drugs CP: 4(RH_3)	100%	Yes Spoligo and MIRU-VNTR
Swaminathan AJRCCM 2010 [10]	Chenai, India Feb '01- Sep '05	100%	0%	IP: Regimen 1) 2($RHEZ_3$) Regimen 2) 2($RHEZ_3$) CP: Regimen 1) 4(RH_3) Regimen 2) 7(RH_3)	DOT was given during IP. 1/3 doses was given as DOT during CP	Yes IS6110, MIRU-VNTR, Spoligo
TB Research Centre IJTLD 1997 [18]	Chennai not specified	Not specified	Not specified	IP: Regimen 1) 2($HREZ_7$) Regimen 2) 2($HREZ_2$) Regimen 3) 2(HRZ_2)CP: Regimen 1) 6(HE_7) Regimen 2) 4(HRE_2) Regimen 3) 4(HR_2)	Regimen 1 was fully unsupervised. Regimen 2 and 3 were either fully or partially supervised.	No
Vernon Lancet 1999 [8]	USA Apr 1995- early 1997	100%	47.5%	IP: 2($RHEZ_{7/3/2}$) CP: Regimen 1) 4(rifapentine/ H_1) Regimen 2) 4(RH_2)	100%	Yes IS6110
Prospective cohorts						
Aung, IJTLD 2012 [19] *operational study with randomisation	Bangladesh Jan '06- Jun '07	Not specified	0%	IP: 2(3) $RHEZ_7$ CP: 4(HR_3)	100%	Yes Sequencing of core region of *rpoB* gene
Burman AJRCCM 2006 [9]	New York City, USA Dec 1998- Mar 2002	100%	Not specified	IP: First 2 weeks: ($RHEZ_7$) Next 6 weeks: ($RHEZ_5$) or ($RHEZ_3$) or ($RHEZ_2$) (78% received rifampin in IP) CP: 4-7(RH_2) R = rifabutin	100%	Yes Sequencing of core region of *rpoB* gene
Cox, Clin Infect Dis 2007 [20]	Karakalpakstan, Uzbekistan and Dashoguz, Turkmenistan Jul 2001- Mar 2002	Not specified	45%	IP: New 2($HREZ_7$) ReRx 2 (SRHEZ7),1($RHEZ_7$) CP: New: 4(HR_3) ReRx:5(HRE_3)	DOT during IP	Yes RFLP of IS6110 and spoligo
El Sahly, J of Infect, 2006 [21]	Houston, USA 1995–2001	18.1%	6.3%	*Not specified	Not specified	Yes RFLP of IS6110 and spoligo
Murray SAMJ 2000 [22]	Goldmines in Gauteng, South Africa, 1995	49%	27%	IP: 2RHZE CP: 4RH	DOT if smear+	No

(*Continued*)

Table 1. (*Continued*)

Reference	Study location and year	HIV prevalence	Retreatment (ReRx)	Regimen	DOT	Genotyping carried out in a proportion of available isolates
Nettles, Clin Infect Dis 2004 [23]	Baltimore, USA Jan '93- Dec '01	27%	Not specified	IP: 2wks ($RHEZ_7$) 6wks ($RHEZ_2$) Rifampicin or rifabutin CP: ($RHEZ_2$) Rifampicin or rifabutin, duration individualised	100%	Yes RFLP of IS6110
Pasipanodya, J Inf Dis 2013 [24]	Western Cape, South Africa	10%	64%	IP: New 2($HREZ_7$) ReRx 2 (SRHEZ7),1($RHEZ_7$) CP: New: 4(HR_3) ReRx:5(HRE_3)	DOT during IP	No
Temple Clin Infect Dis 2008 [14]	Kampala, Uganda Jul 2003- Nov 2006	48%	100%	IP: 1($SRHEZ_7$) 2($RHEZ_7$) CP: 5(RHE_7)	DOT in IP (hospitalised)	Yes RFLP of IS6110
Retrospective cohorts						
Chien, JAC 2013 [25]	Taiwan 2005 to 2011	0%	Not specified	WHO recommendations IP: New 2($HREZ_7$) ReRx 2 (SRHEZ7),1($RHEZ_7$) CP: New 4(HR_3) ReRx 5(HRE_3)	57% received DOTS	No
Driver, Clin Infect Dis, 2001 [26]	New York City Jan 1993- Jun 1996	33%, (unknown 36%)	0%	IP: Regimen 1) Regimen 2 (RHZ_7) Regimen 2) 2 (RHZ_7) Regimen 3) IP with < 8weeks of Z CP: Regimen 1) 4(RH_7) Regimen 2) 6(HE_7) Regimen 3) 7(RH_7)	DOT median 21 weeks	Yes RFLP of IS6110
Gelmanova, Bull WHO, 2007 [27]	Tomsk, Siberia Jan 2001- Dec2001	1%	Not specified	WHO recommendations IP: New) 2($HREZ_7$) ReRx): 2 (SRHEZ7),1($RHEZ_7$) CP: New: 4(HR_3) ReRx:5(HRE_3)	DOT in inpatient, outpatient and home care setting. Small proportion self-administered therapy	No
Jasmer, AJRCCM, 2004 [28]	San Francisco, United States 1998 to 2000	13%	9%	*Not specified	DOT (n = 149) and SAT (n = 223)	No
Kim BMC ID 2008 [13]	Seoul, Korea Jul 2001-Jun 2005.	36%	Not specified	IP: 2($RHEZ_7$) CP: 33% 4 (REZ_7) 54% 10(RE_7) 13% 7 (RE_7)	Not specified	No
Li CID 2005 [29]	New York City Jan 1997 –Dec 2000	Not specified	28%	IP: Variable rifampin or rifabutin-based regimen, daily or intermittent dose (2/ wk or 3/wk) for 2 months CP: rifampin or rifabutin regimen given x2 or 3/wk for 4–6, 7–10 or >10 months	Not specified	Yes RFLP of IS6110 and spoligo
Matthys, PLoS ONE, 2009 [11]	Mariinsk, Siberia, Russia 1997 to 1998	None at entry into prison	65%	IP: 2($SRHEZ_7$),1($RHEZ_7$) CP: 5(RHE_7)	100%	Yes RFLP of IS6110
Moulding IJTLD 2004 [30]	Los Angeles, US Jun 1985-Jul 1992	Cohort known or presumed to be HIV negative	Not specified	IP: HR and Z or E or ZE (duration and frequency not specified) CP: HR (duration and frequency not specified)	Not specified	No
Porco CID 2012 [31]	California, USA Jan 1994- Dec 2006	7.5%	Not specified	*Not specified	100%	No

(*Continued*)

Table 1. (*Continued*)

Reference	Study location and year	HIV prevalence	Retreatment (ReRx)	Regimen	DOT	Genotyping carried out in a proportion of available isolates
Quy IJTLD 2003 [32]	Ho Chi Minh City, Vietnam Aug 1996-Jul 1998	Not specified	0%	IP: New: 2(SHRZ$_7$) ReRx 2 (SRHEZ$_7$),1(RHEZ$_7$) CP: New: 6(HE$_7$) ReRx:5(HRE$_3$)	100%	Yes RFLP of IS6110
Seung CID 2004 [33]	Tomsk, Siberia Nov 1996- Dec 2000	Not specified	0%	IP: 2(HREZ$_7$) In some cases S was given instead of E CP: 4(HR$_7$)	DOT programme IP- hospitalised CP- outpatient	No
Spellman 1988 AIDS [34]	Miami and New York, USA Jan '88- Dec '95	12.8	5.2%	*Not specified	100%	No
Weis, NEJM 1994 [35] #Although presented under cohort studies, this may also be classified as an ecological study	United States 1980 to 1992	58 amongst 485 those tested from 1987 (12%)	Not specified	IP: 1980 to 1986 included HRE. 1986 to 1992 included HRZ +/- E or injectable CP: Not specified	Until 1986 not DOT, from 1986 90.5% received DOT	No
Yoshiyama IJTLD 2004 [15]	Chiang Rai, Thailand May 1996- Dec 2000	31%	100% of re-registered cohort	IP: 2(SRHEZ$_7$),1(RHEZ$_7$) CP: 5(RHE$_7$)	DOT introduced in 1996	Yes RFLP of IS6110
Yuen, PLoSONE 2013 [36]	United States 2004 to 2011	Positive 7% Negative 67.5% Unknown 25.5%	0%	*Not specified	DOT only 61%, DOT + SAT 37%, SAT only 2%	No
Case controls						
Bradford Lancet 1996 [37]	San Fransciso, USA Jan '85-Dec '94	Cases 79% Controls 27%	Cases 14% Controls 14%	*Not specified	Not specified	Yes RFLP of IS6110
Munsiff, Clin Infect Dis 1997 [38]	New York City, USA 93–94	100%	Not specified	IP: Regimen contained RHZ (+/-E), dosing regimen not specified CP: *Not specified	Cases: 24% received DOT Controls: 31% received DOT	No
Weiner CID 2005 [39]	New York City Dec 1998- Mar 2002	100%	Not specified	IP: First 2 weeks: (RHEZ$_7$) Next 6 weeks: (RHEZ$_5$) or (RHEZ$_3$) or (RHEZ$_2$) CP: 9 (RH$_2$)	100%	Yes Sequencing of core region of *rpoB* gene

Abbreviations: IP intensive phase CP continuation phase R rifampin H isoniazid E ethambutol Z pyrazinamide S streptomycin Rx treatment wk week DOT directly observed therapy SAT self-administered therapy X(RHEZ$_Y$) X = number of months on regimen y = number of days/week on regimen ARR acquired rifamycin resistance Spoligo Spoligotyping MIRU-VNTR (mycobacterial interspersed repetitive unit-variable- number tandem repeat) typing RFLP of IS6110 restriction fragment length polymorphism of the IS6110 insertion element

*individualised treatment as per Centre of Disease Control, USA guidelines http://www.cdc.gov/mmwr/pdf/rr/rr5211.pdf.

Whilst treatment regimens were not explicitly stated in 6 (19%) of studies, these all included treatment with a rifamycin during intensive phase and were of minimum 6 months duration.

doi:10.1371/journal.pone.0139017.t001

with patients with baseline pan-susceptible MTB (Fig 6). There was moderate heterogeneity of the data as evidenced by I^2 58% (95% CI 26 to 76%), the same positive trend was seen in all 15 studies included.

A funnel plot for the meta-analysis of baseline drug resistance as a risk factor for ADR (Fig 7) showed a dearth of smaller studies reporting negative effects. However, the asymmetry of the funnel plot also appears to be related to substantial heterogeneity among the larger studies

Reference	Cohort description and numbers	Acquired isoniazid resistance (%)	Acquired rifamycin resistance (%)	Acquired MDR TB (%)	Risk factors associated with acquired drug resistance (ADR)
**Algerian Working Group/British MRC 1991 Tubercle [16]**	Whole cohort n=2218<br>Known baseline drug sensitivity n=2071<br>Follow up drug sensitivity n=1415	WC= 1/2071 (0.05)<br>WCFU= 1/1415 (0.07)<br>PS= 1/1376 (0.07)<br>$MR_{(S)}$ = 0/40 (0)	WC= 4/2071 (0.19)<br>WCFU= 4/1415 (0.28)<br>PS= 1/1376 (0.07)<br>$MR_{(H/S)}$=0/61 (0)<br>PR $_{(H+S)}$= 3/34 (8.8)	WC= 4/2071 (0.19)<br>WCFU= 4/1415 (0.28)<br>PS=1/1376 (0.07)<br>$MR_{(H /S)}$=0/50<br>$PR_{(H+S)}$ = 3/33 (9)	-Rifampicin in regimen only during intensive phase<br>-Baseline resistance to INH and STREP
**Hong Kong TB Research Centre Madras/BMRC Am Rev Resp Disease 1991 [12]**	Whole cohort n=145 Culture proven TB with known baseline sensitivity n=127	WCFU 2/127 (1.6)<br>PS= 2/91 (2.2)<br>$MR_{(S/R)}$= 0/13 (0)	WCFU 5/127 (3.9)<br>PS 1/91 (1.1)<br>$MR_{(H/S)}$ = 1/22 (4.5)<br>PR= 3/9 (33)	WC= 4/127 (3.1)<br>PS= 1/91 (1.1)<br>$MR_{(H/S/R)}$= 1/25 (4)<br>$PR_{(H+S)}$= 3/9 (33)	-Baseline drug resistance
**Lienhardt JAMA 2011 [17]**	Culture confirmed smear +ve new TB patients either pan-susceptible or INH monoresistant n=1170	WCFU 1/1170 (0.09)<br>PS 1/1005 (0.1)	WCFU 1/1170 (0.09)<br>PS 0/1005 (0)<br>$MR_{(H)}$ 1/127 (0.79)	WCFU 1/1170 (0.09)<br>PS 0/1005 (0)<br>$MR_{(H)}$ 1/127 (0.79)	None of the factors analysed were associated<br>(see S4 Table)
**Swaminathan AJRCCM 2010 [10]**	New TB cases (baseline MDR excluded) n=327 Culture confirmed with DST and results at end of treatment	WC 7/327 (2)<br>WCFU 7/212 (3.3)<br>PS 7/194 (3.6)	WC 20/327 (6.1)<br>WCFU 20/212 (9.4)<br>PS 11/194 (5.7)	WC 17/327 (5.2)<br>WCFU 17/212(8.0)<br>PS 11/194 (5.7)	-Lower median CD4 lymphocyte count (p 0.054)<br>- Higher median HIV VL (p 0.009)<br>Non-adherence (adherence <90%) (p 0.000)

Fig 2. RCTS- ADR and associated risk factors.

doi:10.1371/journal.pone.0139017.g002

with small standard errors, around the summary estimate of effect, with a resulting imbalance toward a large positive effect estimate.

ADR was significantly associated with extrapulmonary/disseminated TB in 3/5 (60%) studies; with smear positivity in 4/8 (50%) studies; and with extensive radiological disease and cavitatory disease in 1/4 (25%) and 1/7 (14%) studies respectively. *M. tuberculosis* complex strain was a risk factor for ADR in 2/4 (50%) studies that examined its role: 1 found increased risk with Beijing strains and 1 with *M. bovis*.

Host immunity and PK variability. HIV co-infection was a risk factor for ADR in 8/10 (80%) studies that assessed it. In a meta-analysis of 8/10 studies (35,595 patients), HIV was a significant risk factor for ADR (RR 3.02, 95% CI 1.28 to 7.11) with overall high heterogeneity I^2 81% (95% CI 64 to 90%) (Fig 8). Sub-group analysis by continent for ADR showed a RR of 3.23 (95% CI 1.02 to 10.26) with HIV co-infection in 5 North American studies (heterogeneity I^2 29%, 95% CI 0 to 72%) whilst there was a trend towards a negative association in 2 African studies (RR 0.3, 95% CI 0.07 to 1.19) with heterogeneity I^2 12%.

A funnel plot for the meta-analysis of HIV as risk factor of ADR indicated little risk of publication bias (Fig 9).

A low CD4 lymphocyte count at diagnosis in 5/8 studies (63%) and an AIDS diagnosis in 2/2 studies were significant risk factors for ADR amongst HIV-infected patients. Gastrointestinal symptoms at baseline were associated with ADR in 1/1 study and concurrent use of antifungal azoles in 2/2 studies. PK variability was found to be a risk factor for ADR in both studies examining its role. Weiner *et al* found that a lower area under the curve ($AUC_{0\text{-}24hr}$) and lower peak concentration (C_{max}) for rifabutin was associated with increased risk of ADR. This was in a

Reference	Cohort description and numbers	Acquired isoniazid resistance (%)	Acquired rifamycin resistance (%)	Acquired MDR TB (%)	Risk factors associated with ADR
**Aung, IJTLD 2012 [19] *operational study with randomisation**	Patients who were smear -ve at 2mth and whose IP was not extended n=12967 Patients who were smear +ve at 2mth and whose IP was extended by 1 mth n=1871 Patients who were smear +ve at 2mth and whose IP was not extended n=1870 Smear defined relapses/failures n=595	Not specified	WC 16/16708 (0.09) WCFU 16/595 (2.7)	WC 12/16708 (0.07) WCFU 12/595 (2)	None of the factors analysed were associated (see S4 Table)
**Burman AJRCCM 2006 [9]**	Culture confirmed TB, HIV co-infection and on intermittent rifabutin* based regimen n=169 *78% initially received a median of 33.5 days of rifampin based therapy during IP	Not specified	WC=8/169 (4.7)	WC=1/169 (0.59)	-Lack of use of ART in first 2 months TB treatment (p=0.05) - Lower CD4 lymphocyte count at diagnosis (p=0.001)
**Cox, Clin Infect Dis 2007 [20]**	Smear +ve TB (baseline MDR patients and mixed infection excluded) n=314, smear +ve with identical spoligotype as baseline at end of IP or 2 months into CP n=62	WCFU 1/314 (0.3) PS 1/177 (0.6) $MR_{(R/S/Z/E)}$ 0/51 (0)	WCFU 11/314 (3.5) PS 1/177 (0.6) $MR_{(I/S/Z/E)}$ 0/71 (0) $PR_{(H+S\ or\ I+S+E\ or\ I+S+E+Z)}$	WCFU 11/314 (3.5) PS 1/177 (0.6) $MR_{(I/S/R/Z/E)}$ 0/72 (0) $PR_{(I+S\ or\ I+S+E\ or\ I+S+E+Z)}$	-Baseline polyresistance (p<0.05) - Beijing genotype in polyresistant strain (11 out of 28 polyresistant Beijing strains amplified their resistance, compared with none of the 27 non-Beijing strains)

Fig 3. Prospective cohorts- ADR and associated risk factors.

doi:10.1371/journal.pone.0139017.g003

sub-cohort of patients who were sampled during continuation phase therapy. There was no significant difference in isoniazid C_{max} or $AUC_{0\text{-}24hr}$ in cases of ADR, compared with controls. Pasipanodya *et al* found that low rifampicin and isoniazid peak concentrations and $AUC_{0\text{-}24hr}$ preceded ADR in 3 patients.

Sociodemographic factors. Older age [4/11(36%)], foreign birth [1/3 (33%)], ethnicity [2/5 (40%)], unemployment [1/1], substance abuse [2/4 (50%)] and homelessness [1/3 (33%)] were found to be risk factors for ADR in certain studies.

TB regimen and adherence. Non-adherence was assessed as a risk factor for ADR in 5/32 studies and was associated with ADR in 3/5 (60%) of studies. Directly observed therapy was a risk factor for ADR in 1/8 (12.5%) studies that compared the practice of SAT with DOT. In contrast, SAT was found to be a risk factor for ADR in 4/8 (50%) studies. There was no association between DOT or SAT and ADR in 3 studies. Separate drug formulation, as opposed to fixed dose combination (FDC), was found to be a risk factor for ADR in 1/3 (33%) of studies. Use of rifampicin in the regimen only during intensive phase [16] and lack of ethambutol [18] in a twice/once weekly dosing regimen were associated with cases of ADR in individual RCTs carried out in the 1990s. In one retrospective cohort study, in a sub-analysis of HIV co-infected patients, intermittent dosing of rifampicin during the intensive phase and use of rifampicin instead of rifabutin was associated with ADR [29]. This was in contrast to sub-analysis of HIV-infected patients in another study where there was no significant difference in ADR comparing rifampicin and rifabutin-based regimens [23]. In a RCT, a once weekly rifapentine based regimen in continuation phase was associated with ADR in HIV co-infected individuals [9].

Reference	Cohort description and numbers	Acquired isoniazid resistance (%)	Acquired rifamycin resistance (%)	Acquired MDR TB (%)	Risk factors associated with ADR
**Chien, JAC 2013 [25]**	Culture confirmed pulmonary TB without HIV co-infection (baseline MDR/XDR excluded) n=2080	WCFU 108/2080 (5.2)	WCFU 160/2080 (4.7)	WCFU 178/2080 (8.6)	-Age group 45-64 (OR 2.07 , p=0.01)<br>-Smear positivity (OR 2.09, , p=0.01)<br>- Self-administration of treatment/lack of DOT (OR 2.94, p=0.01)
**Driver, Clin Infect Dis, 2001 [26]**	Drug sensitive at baseline n=4571, Known relapses/recurrences at end of at least 6mths Rx n=123, Known DST at relapse or recurrence n=95	WCFU 14/4571 (0.3)<br>PS 14/4571 (0.3)	WCFU 21/4571 (0.5)<br>PS 21/4571 (0.5)	WCFU 9/4571 (0.2)<br>PS 9/4571 (0.2)	-HIV co-infection (risk factor for acquired RIF monoresistance)
**Gelmanova , Bull WHO, 2007 [27]**	Enrolled in DOT n=260 , Culture +ve, with known DST (baseline MDR at excluded) n=207	Not specified	Not specified	WCFU 15/207 (7.3)	-Substance abuse (HR 2.88, p=0.04)<br>-Treatment commenced in hospital setting (HR=6.34, p=0.02)<br>- Hospitalisation later in treatment (HR=6.26 ,p=0.047)<br>-Self-administration of Rx/lack of DOT (HR 0.25, p=0.03)
**Jasmer, AJRCCM, 2004 [28]**	Drug sensitive cases of TB who started treatment with allocated DOT status n=372,	WC 2/372 (0.5)<br>WCFU 2/330 (0.6)			None of the factors analysed were associated<br>(see S4 Table)

Fig 4. Retrospective cohorts- ADR and associated risk factors.

doi:10.1371/journal.pone.0139017.g004

Cumulative incidence of ADR. Figs 2–5 report DST data and cumulative incidence of acquired isoniazid, rifamycin and MDR for individuals studies stratified by whole cohort, whole cohort with follow up DST, baseline pan-susceptibility, baseline mono-resistance and baseline poly-resistance. In 25 studies, which reported acquired MDR, when considering the overall cohort as denominator, the weighted median incidence of acquired MDR was 0.1% (5th to 95th percentile 0.07 to 3.2%). In 20 studies reporting acquired isoniazid resistance, when considering the overall cohort as denominator, the weighted median incidence of acquired isoniazid resistance was 0.1% (5th to 95th percentile 0.1 to 0.7%). In the 27 studies reporting acquired rifamycin resistance, when considering the overall cohort as denominator, the weighted median incidence of acquired rifamycin resistance was 0.1% (5th to 95th percentile 0.09 to 0.7%). In patients with baseline pan-susceptibility (data available in 15 studies) the weighted median incidence of acquired MDR was 0.2% (5th to 95th percentile 0 to 0.9%). In those with baseline pan-susceptibility, acquired isoniazid resistance (weighted median incidence 0.3%, 5th to 95th percentile 0.06 to 2.7%) did not appear to be more frequent than acquired rifamycin resistance (weighted median incidence 0.3%, 5th to 95th percentile 0 to 0.9%). The weighted median incidence of acquired MDR in patients with baseline monoresistance (data available in 12 studies) was 1% (5th to 95th percentile 0.79 to 10%). The weighted median incidence of acquired MDR in patients with baseline polyresistance (data available in 7 studies) was 10% (5th to 95th percentile 7.1 to 15.5%). It must be noted, that the above estimates of incidence of ADR refer only to studies included in this review and with our search strategy, we excluded studies in which no cases of ADR occurred.

Reference	Cohort description and numbers	Acquired isoniazid resistance (%)	Acquired rifamycin resistance (%)	Acquired MDR TB (%)	Risk factors associated with ADR
**Bradford Lancet 1996 [37]**	Total TB cases reported with known DST n=2612 Cases: acquired resistance to R,H or E with baseline pan-susceptibility n= 14 Control: baseline pan-susceptibility, no ADR, matched to time of diagnosis as cases n=56	WC 7/2612 (0.3)	WC 10/2612 (0.3)	WC 3/2612 (0.1)	- White ethnicity (p=0.015)<br>-Foreign birth (p=0.007)<br>-Unemployment (p=0.017)<br>- Self-administration of treatment/lack of DOT (p=0.045)<br>-ART use (p=0·014)<br>-Azole use (p<0·001)<br>-GI symtoms (aOR=11.5, 95%CI=1.23-107)<br>-Non-adherence (aOR=19.7, 95%CI=1.66-234.4)<br>-Baseline AIDS (aOR=20.2, 95%CI=1.12-363.6)<br>aOR adjusted odds ratio
**Munsiff, Clin Infect Dis 1997 [38]**	Cases: HIV-TB co-infected patients with confirmed acquired rifamycin monoresistance n=29 Control: HIV-TB co-infected patients with drug sensitive TB n=58	N/A	N/A	N/A	-Non-adherence (OR 11.0, p<0.001)<br>-Baseline AIDS (OR 5.6, p=0.005])<br>-Baseline smear positivity (OR= 4.1, p=0.02)

Fig 5. Case-Control studies- ADR and associated risk factors.

doi:10.1371/journal.pone.0139017.g005

Discussion

Although acquired MDR was rare overall [weighted median frequency 0.1%], it was more frequent in certain risk groups such as those with baseline mono or polyresistance. A meta-analysis of 15 studies with a moderately heterogeneous data set showed a RR for ADR of 4.96 in patients with baseline drug resistance compared with baseline pan-susceptible profiles. Studies reporting ADR as a treatment outcome varied in geographical location, HIV co-infection, retreatment proportions and treatment regimens administered during intensive and continuation phase as summarised in Table 1 and S2 Table. Weighted pooled analysis of a highly heterogeneous data set showed an increased risk of ADR (RR 3.02) with HIV co-infection. The data presented disaggregated by continent showed a significant association with HIV co-infection in 5 North American studies whilst there was a trend towards a negative association in 2 African studies. This negative association of HIV with ADR in Africa, may partly be explained by a relatively higher proportion of HIV infected patients who develop ADR dying prior to the detection of ADR. Advanced immunosuppression as reflected by a lower baseline CD4 lymphocyte count or AIDS at diagnosis was a risk factor in HIV co-infected patients. Poor adherence and extrapulmonary/disseminated disease were risk factors for ADR in 60% of studies. There was less conclusive evidence regarding the role of PK variability, strain type, DOT versus self-administered therapy, fixed dose combinations and choice of rifamycin as risk factors.

The wide range in reported incidence of ADR may be partially explained by lack of standardization in reporting. For example, where follow up culture and DST results are missing, either the denominator can be altered to reflect this, or the denominator remains as the original cohort number; the assumption being that those with missing DST did not develop ADR. In

Study or Subgroup	Baseline drug resistance Events	Baseline drug resistance Total	Pan-susceptible Events	Pan-susceptible Total	Weight	Risk Ratio M-H, Random, 95% CI
**1.2.1 Africa**						
Algerian Working Group/British MRC 1991	3	95	1	1376	2.6%	43.45 [4.56, 413.75]
Murray 2000	3	50	9	350	5.9%	2.33 [0.65, 8.33]
Temple 2008	3	31	5	226	5.4%	4.37 [1.10, 17.41]
**Subtotal (95% CI)**		**176**		**1952**	**13.9%**	**5.87 [1.41, 24.41]**
Total events	9		15			
Heterogeneity: Tau² = 0.93; Chi² = 4.91, df = 2 (P = 0.09); I² = 59%						
Test for overall effect: Z = 2.43 (P = 0.01)						
**1.2.2 North America**						
Burman 2006	1	8	7	161	3.2%	2.88 [0.40, 20.64]
Porco 2012	33	3177	67	30548	12.9%	4.74 [3.13, 7.17]
Spellman 1998	2	57	3	682	3.8%	7.98 [1.36, 46.77]
Yuen 2013	23	348	90	3672	12.7%	2.70 [1.73, 4.21]
**Subtotal (95% CI)**		**3590**		**35063**	**32.6%**	**3.72 [2.48, 5.58]**
Total events	59		167			
Heterogeneity: Tau² = 0.05; Chi² = 4.10, df = 3 (P = 0.25); I² = 27%						
Test for overall effect: Z = 6.36 (P < 0.00001)						
**1.2.3 Asia**						
Cox 2007	10	82	1	177	3.0%	21.59 [2.81, 165.82]
Hong Kong TB Research 1991	4	34	3	91	5.1%	3.57 [0.84, 15.13]
Quy 2003	13	116	2	133	4.9%	7.45 [1.72, 32.34]
Seung 2004	25	398	12	1212	10.5%	6.34 [3.22, 12.51]
Swaminathan 2010	9	26	12	194	9.7%	5.60 [2.61, 11.98]
TB Research Centre 1997	24	228	25	387	11.8%	1.63 [0.95, 2.78]
Yoshiyama 2004	11	237	4	1634	6.8%	18.96 [6.09, 59.06]
**Subtotal (95% CI)**		**1121**		**3828**	**51.8%**	**5.87 [2.82, 12.21]**
Total events	96		59			
Heterogeneity: Tau² = 0.65; Chi² = 23.84, df = 6 (P = 0.0006); I² = 75%						
Test for overall effect: Z = 4.73 (P < 0.00001)						
**1.2.4 Europe**						
Matthys 2009	6	108	0	81	1.7%	9.78 [0.56, 171.14]
**Subtotal (95% CI)**		**108**		**81**	**1.7%**	**9.78 [0.56, 171.14]**
Total events	6		0			
Heterogeneity: Not applicable						
Test for overall effect: Z = 1.56 (P = 0.12)						
**Total (95% CI)**		**4995**		**40924**	**100.0%**	**4.85 [3.26, 7.23]**
Total events	170		241			
Heterogeneity: Tau² = 0.27; Chi² = 33.61, df = 14 (P = 0.002); I² = 58%						
Test for overall effect: Z = 7.77 (P < 0.00001)						
Test for subgroup differences: Chi² = 1.68, df = 3 (P = 0.64), I² = 0%						

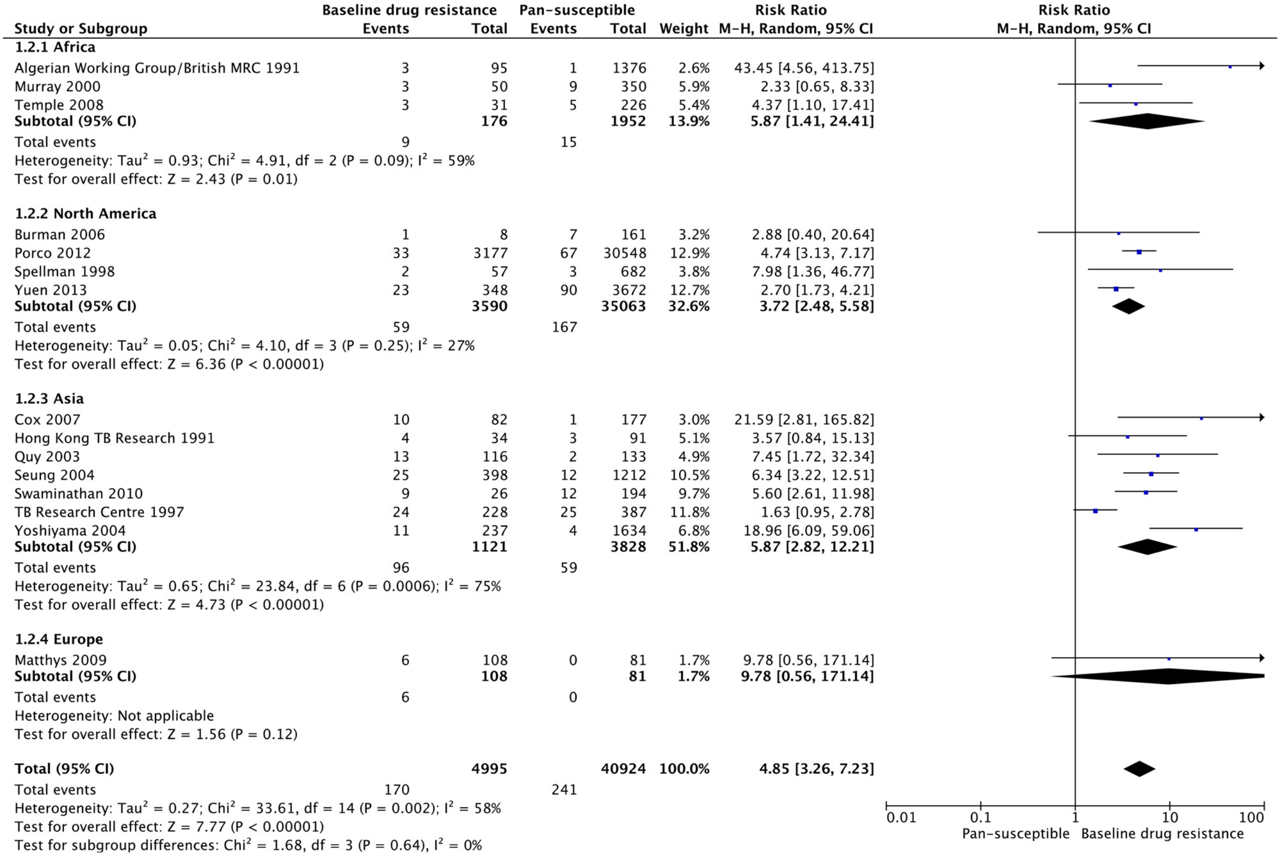

Fig 6. Forest plot of comparison: 1) baseline drug resistance vs pansusceptible MTB, outcome of ADR: 1.2) ADR by region. * 1 study was excluded as we were unable to obtain the exact proportion of patients in the study with non-MDR baseline drug resistance and baseline pan-susceptibility either from the paper or by contacting the authors. The endpoint used for the plot for 12 studies was acquisition of isoniazid/rifamycin/multidrug resistance [3,9,11,13,14,17,19,21,30,31,32,33] and the end point for 3 studies was acquisition of rifamycin resistance [8,28,35], based on data available.

doi:10.1371/journal.pone.0139017.g006

this review we have presented cumulative incidence of ADR in individual studies, for both the whole cohort and limited to those with follow up DST data.

Baseline mono or polyresistance has previously been recognised as a significant risk factor for ADR. Lew *et al* [40] carried out a meta-analysis looking at the role of initial drug resistance on TB treatment outcomes. Of note, many studies carried out in the 1970s only used rifamycins during a 2 month intensive phase. Lew *et al* found that the cumulative incidence of ADR increased from 0.8% (95% CI 0.5 to 1%) in baseline pan-susceptible cases to 6% (95% CI 4 to 8%) in baseline monoresistant cases and 14% (95% CI 9 to 20%) in baseline polyresistant cases [40]. A review by Menzies *et al* [41] found that in patients with baseline isoniazid monoresistance, a longer duration of rifampicin, use of streptomycin, daily therapy initially, and treatment with a greater number of effective drugs were associated with reduced risk of ADR. Jacobsen *et al* reported 9% progression to MDR TB in a cohort with baseline isoniazid monoresistance who received 12 months of quadruple therapy [42]. In many resource limited settings, Xpert MTB/RIF is used to test for baseline rifampicin resistance and baseline isoniazid resistance will go undetected. During continuation phase, those with isoniazid monoresistance (particularly high level) who are still culture positive, will be effectively receiving rifampicin monotherapy. Hence, there is potential for amplification of drug resistance.

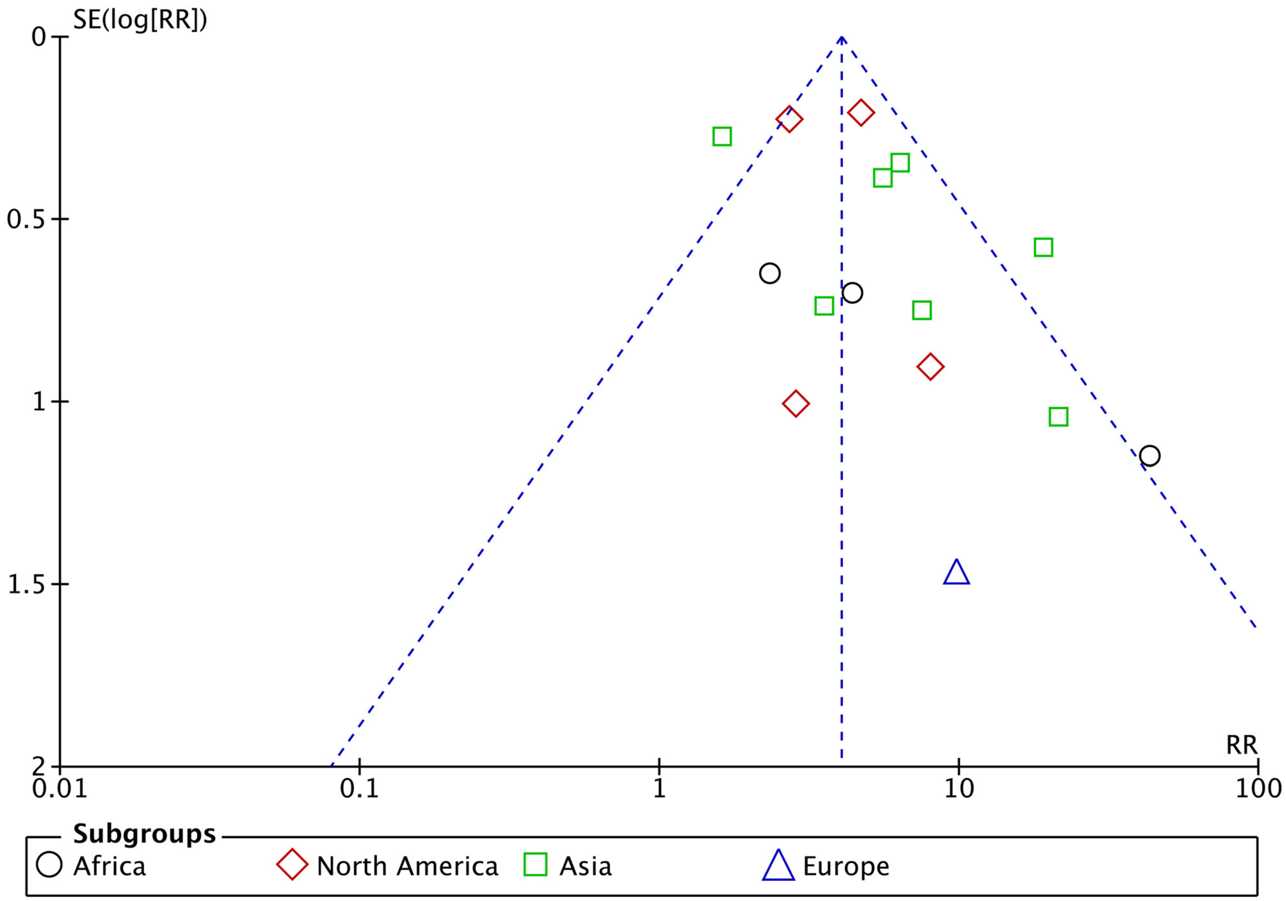

Fig 7. Funnel plot of studies included in meta-analysis of baseline drug resistance and ADR.

doi:10.1371/journal.pone.0139017.g007

The role of HIV co-infection in the acquisition of TB drug resistance has been a topic of debate. In an immunocompromised host, there is an increased risk of disseminated TB; the latter being an independent risk factor for ADR. Hence, there may be an increased bacterial burden leading to an increased probability of bacteria undergoing spontaneous mutation. It is also hypothesized that less fit drug resistant strains survive longer in the context of poor immunity, allowing for development of compensatory mutations to restore fitness [43–45]. Some MTB strain types are particularly prevalent in immunocompromised hosts [45]. HIV co-infection may cause changes in gut permeability leading to malabsorption of antituberculous drugs [5,46]. As antiretroviral therapy (ART) becomes increasingly available and guidelines advocate early commencement of ART, it remains to be seen if HIV co-infection will continue to be associated with ADR.

In vitro work in hollow fibre models has suggested that PK variability and inadequate dosing of TB drugs may be an important risk factor for ADR [47,48]. This is supported by findings from cohort studies [24,39]. However, these results need to be confirmed in studies with robust determination of PK indices and appropriate controls. Pasipanodya *et al* [49] reviewed the role of N-acetyl-transferase type 2 genotype in acquired isoniazid resistance. The link they found between slow acetylator status and ADR may be less significant in the context of currently utilised rifampicin-containing multidrug regimens.

Study or Subgroup	HIV positive Events	HIV positive Total	HIV Negative Events	HIV Negative Total	Weight	Risk Ratio M-H, Random, 95% CI
**1.1.1 N. America**						
Li 2005	7	807	3	2054	13.0%	5.94 [1.54, 22.91]
Nettles 2004	3	108	0	161	5.8%	10.40 [0.54, 199.41]
Porco 2012	35	2330	65	29603	18.4%	6.84 [4.55, 10.30]
Spellman 1998	1	95	5	644	8.7%	1.36 [0.16, 11.48]
Yuen 2013	24	275	19	2712	17.6%	12.46 [6.91, 22.45]
**Subtotal (95% CI)**		**3615**		**35174**	**63.5%**	**7.77 [4.81, 12.53]**
Total events	70		92			
Heterogeneity: $Tau^2 = 0.08$; $Chi^2 = 5.63$, df = 4 (P = 0.23); $I^2 = 29\%$						
Test for overall effect: Z = 8.40 (P < 0.00001)						
**1.1.2 Africa**						
Murray 2000	1	196	8	201	9.0%	0.13 [0.02, 1.02]
Temple 2008	2	173	4	183	11.0%	0.53 [0.10, 2.85]
**Subtotal (95% CI)**		**369**		**384**	**20.1%**	**0.30 [0.07, 1.19]**
Total events	3		12			
Heterogeneity: $Tau^2 = 0.12$; $Chi^2 = 1.13$, df = 1 (P = 0.29); $I^2 = 12\%$						
Test for overall effect: Z = 1.71 (P = 0.09)						
**1.1.3 Asia**						
Yoshiyama 2004	9	17	7	37	16.4%	2.80 [1.25, 6.25]
**Subtotal (95% CI)**		**17**		**37**	**16.4%**	**2.80 [1.25, 6.25]**
Total events	9		7			
Heterogeneity: Not applicable						
Test for overall effect: Z = 2.51 (P = 0.01)						
**Total (95% CI)**		**4001**		**35595**	**100.0%**	**3.02 [1.28, 7.11]**
Total events	82		111			
Heterogeneity: $Tau^2 = 1.00$; $Chi^2 = 37.31$, df = 7 (P < 0.00001); $I^2 = 81\%$						
Test for overall effect: Z = 2.53 (P = 0.01)						
Test for subgroup differences: $Chi^2 = 20.93$, df = 2 (P < 0.0001), $I^2 = 90.4\%$						

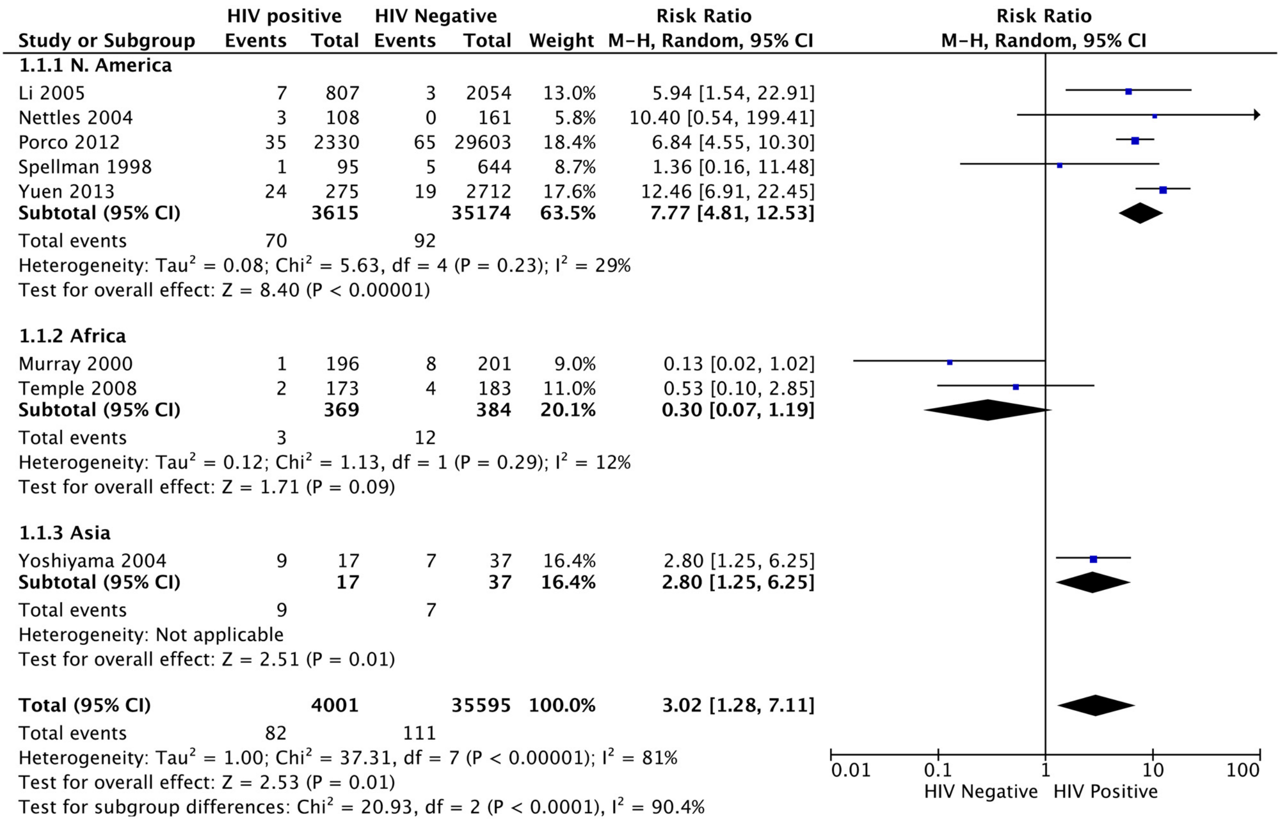

Fig 8. Forest plot of comparison: 1) HIV status, outcome of ADR. 1.2) ADR by region. Only 8/10 studies which examined HIV as a risk factor for ADR were included as we were unable to obtain the exact proportion of those HIV seropositive among the patients that developed ADR from either the paper or by contacting the authors in the other 2 studies. The end point for 5 studies was acquisition of isoniazid/rifamycin/multidrug resistance [13,14,21,30,33] and the end point for 3 studies was acquisition of rifamycin resistance [22,28,35], based on data available.

doi:10.1371/journal.pone.0139017.g008

Two studies showed an association with MTB strain. Cox *et al* found an association between Beijing strain and ADR [20]. In a database of 3696 MTB complex strains, 72% of which were Euro-American lineage, only *M*. *bovis* was associated with ADR [36]. Luria-Delbrück fluctuation analyses have suggested that MTB lineage 2 (Beijing) strains are associated with increased mutation rates and acquisition of drug resistance [50]. This may potentially be through sign epistasis where there is favourable interaction between drug resistance mutations and genetic background of the strain [51].

There is no standardized means of measuring adherence and the measure chosen depends on the setting, burden of disease, infrastructure and resources available. Whilst some studies have used DOT (as opposed to SAT), as a surrogate measure of adherence, we have not made this assumption as the outcome of DOT may be confounded by its indication. We have examined non-adherence as a separate risk factor to DOT versus SAT. Non-adherence was a significant risk factor in 3/5 [9,36,37] of the studies that examined its association with ADR. For the 2 studies which showed no significant association between non-adherence and ADR, there was a trend of positive association for 1 of the studies [26] but in the other, all 5 cases of ADR were noted to be adherent with therapy. The impact of DOT versus SAT on ADR was less clear with a protective effect of DOT seen in 50% of studies. A meta-analysis by Pasipanodya *et al* showed no increased risk of microbiologic failure, relapse, or ADR with DOT compared with SAT [52].

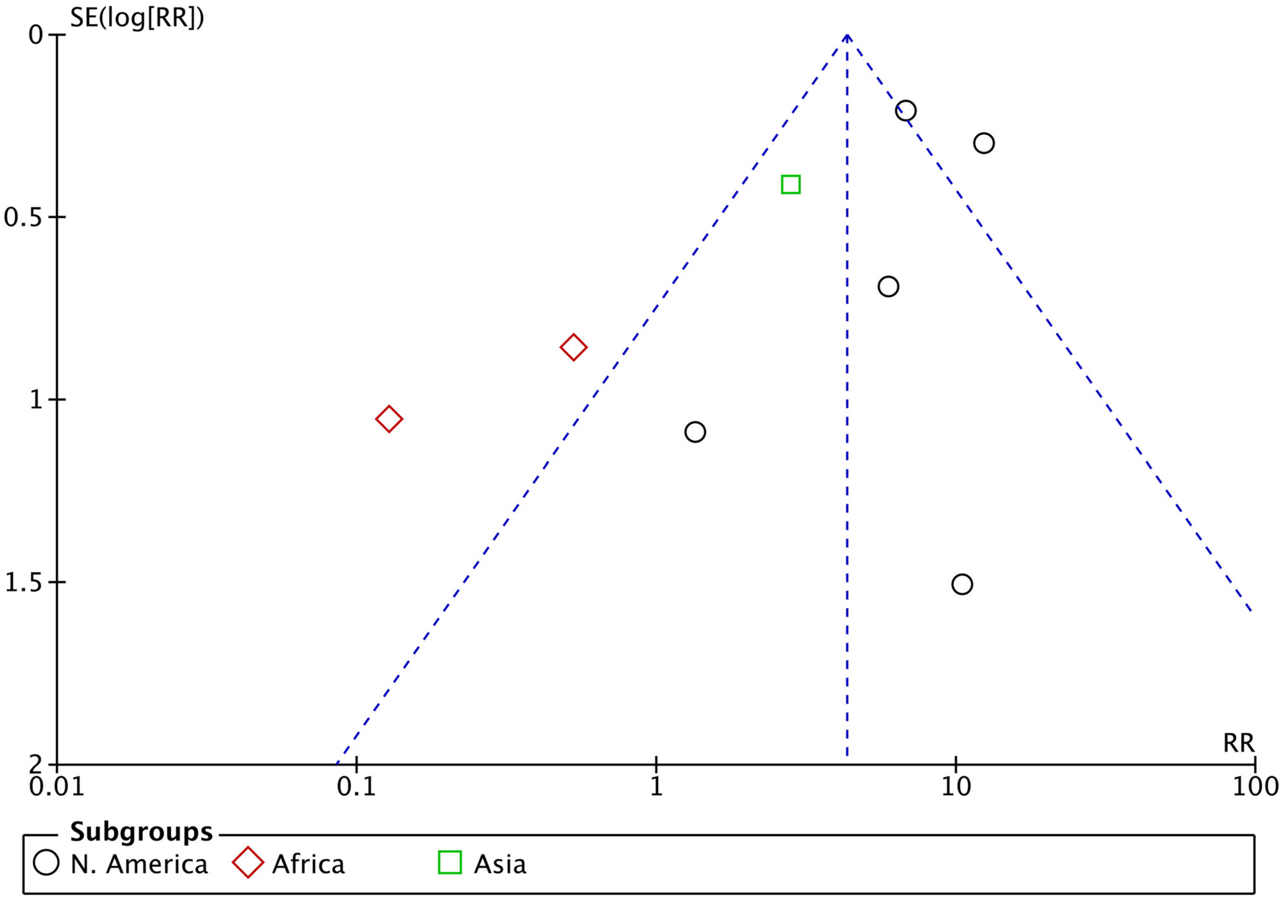

Fig 9. Funnel plot of studies included in meta-analysis of HIV and ADR.

doi:10.1371/journal.pone.0139017.g009

Intermittent dosing frequency has been linked with adverse outcomes, including ADR, when administered during intensive phase [8,29], particularly in the context of HIV co-infection.

There are several limitations to this review. The primary focus of the review was evaluating risk factors for ADR. It is not possible to gather any meaningful data regarding risk factors for an event from a study in which no events are reported and consequently, studies that either reported ADR but no risk factors or 0% ADR were excluded and this potentially affected the estimates of ADR cumulative incidence, which was a secondary analysis. There was incomplete MTB strain genotyping to rule out the possibility of dual mixed infection or exogenous re-infection. Only 47% of studies confirmed ADR with identical MTB genotype at baseline and follow-up. Even where genotyping was part of the study design, in some, a proportion of suspected ADR isolates were not available for genotyping [9,14,25,28]. Many studies were retrospective and had small sample sizes and missing DST. Hence, some studies were likely to have been underpowered and there may have been misclassification bias. There were no statistical analyses of risk factors for ADR in 13 studies because the primary outcome was not ADR. We were limited to noting trends of risk for ADR in the studies. We only conducted meta-analyses of HIV co-infection and baseline drug resistance as risk factors. For the meta-analyses undertaken, the weighted estimates of effect size, must be taken in context of moderate to high heterogeneity in the random effects model [53,54]. This heterogeneity is not surprising, considering different geographical populations, varying MTB strains, different regimens and

dosing frequencies, different programmatic factors such as self-administered vs DOT and different proportions of retreatment vs new patients. There were also differences in study methodology such as choice of denominator in the calculation of cumulative incidence of ADR and lack of confirmatory genotyping in all studies.

Previous reviews have focussed on a specific risk factor such as fixed dose combination vs. separate drug formulation [55], duration and dosing frequency of rifamycin [56] and baseline isoniazid monoresistance[40]. The strength of this review is that it consolidates the multifactorial aetiology of ADR within a single systematic review.

In conclusion, baseline drug resistance and HIV co-infection were significant risk factors for ADR. Overall, there were limitations of the current evidence and difficulties in evaluating possible contributors to ADR with heterogeneity secondary to both clinical and/or methodological diversity. Although the data are variable, disseminated disease and non-adherence had positive trends of association for ADR. There are likely many other variables contributing to acquired rifamycin and/or isoniazid resistance and studies to date have not adequately evaluated factors such as PK variability and MTB strain type as risk factors for ADR. The multifactorial aetiology ADR in a programmatic setting should be further evaluated via appropriately designed studies.

Supporting Information

S1 File. RevMan data.
(RM5)

S1 PRISMA Checklist.
(DOC)

S1 Table. Characteristics of included studies including location and year, criteria for repeat DST and technique used.
(DOCX)

S2 Table. Aggregate data of studies included in the review.
(DOCX)

S3 Table. Study quality based on criteria developed in the Critical Appraisal Skills Programme.
(DOCX)

S4 Table. Risk factors for acquired drug resistance examined.
(DOCX)

Acknowledgments

The authors thank Vittoria Lutje, information retrieval specialist at the Cochrane Infectious Diseases Group, Liverpool, for assistance with carrying out the literature searches, Taryn Young, Centre for Evidence-based Health Care, Stellenbosch University, for methodological guidance, Michael Schomaker, School of Public Health and Family Medicine, University of Cape Town for methodological guidance and Mark Engel, Evidence-Based Medicine Research Support Unit, University of Cape Town for methodological guidance and assistance with designing figures.

Author Contributions

Conceived and designed the experiments: NR GM RJW. Performed the experiments: NR GM. Analyzed the data: NR GM LA. Contributed reagents/materials/analysis tools: NR GM LA. Wrote the paper: NR GM RJW.

References

1. World Health Organisation. Multidrug-resistant tuberculosis (MDR-TB) 2013 Update. 2013. http://www.who.int/tb/challenges/mdr/en/. Updated 2013. Accessed January/20, 2015.
2. Gandhi NR, Nunn P, Dheda K, Schaaf HS, Zignol M, van Soolingen D, et al. Multidrug-resistant and extensively drug-resistant tuberculosis: a threat to global control of tuberculosis. Lancet. 2010; 375 (9728):1830–43. doi: 10.1016/S0140-6736(10)60410-2 PMID: 20488523
3. David HL. Probability distribution of drug-resistant mutants in unselected populations of *Mycobacterium tuberculosis*. Appl. Microbiol.1970 20:810–814. PMID: 4991927
4. Srivastava S, Pasipanodya JG, Meek C, Leff R, Gumbo T. Multidrug-resistant tuberculosis not due to noncompliance but to between-patient pharmacokinetic variability. J Infect Dis 2011; 204(12):1951–9. doi: 10.1093/infdis/jir658 PMID: 22021624
5. Peloquin CA, Berning SE, Huitt GA, Iseman MD. AIDS and TB drug absorption. Int J Tuberc Lung Dis 1999; 3(12):1143–4. PMID: 10599022
6. Muller B, Borrell S, Rose G, Gagneux S. The heterogeneous evolution of multidrug-resistant *Mycobacterium tuberculosis*. Trends Genet: TIG. 2013; 29(3):160–9. doi: 10.1016/j.tig.2012.11.005 PMID: 23245857
7. Critical Appraisal Skills Programme. http://www.sph.nhs.uk/what-we-do/public-healthworkforce/
8. Vernon A, Burman W, Benator D, Khan A, Bozeman L. Acquired rifamycin monoresistance in patients with HIV-related tuberculosis treated with once-weekly rifapentine and isoniazid. Tuberculosis Trials Consortium. Lancet. 1999; 353(9167):1843–7. PMID: 10359410
9. Burman W, Benator D, Vernon A, Khan A, Jones B, Silva C, et al. Acquired rifamycin resistance with twice-weekly treatment of HIV-related tuberculosis. Am J Respir Crit Care Med. 2006; 173(3):350–6. PMID: 16109981
10. Swaminathan S, Narendran G, Venkatesan P, Iliayas S, Santhanakrishnan R, Menon PA, et al. Efficacy of a 6-month versus 9-month Intermittent Treatment Regimen in HIV-infected Patients with Tuberculosis A Randomized Clinical Trial. Am J Respir Crit Care Med. 2010; 181(7):743–51. doi: 10.1164/rccm.200903-0439OC PMID: 19965813
11. Matthys F, Rigouts L, Sizaire V, Vezhnina N, Lecoq M, Golubeva V, et al. Outcomes after chemotherapy with WHO category II regimen in a population with high prevalence of drug resistant tuberculosis. PloS One. 2009; 4(11):e7954. doi: 10.1371/journal.pone.0007954 PMID: 19956770
12. Hong Kong Chest Service Tuberculosis Research Centre MBMRC. A Controlled Clinical Comparison of 6 and 8 Months of Antituberculosis Chemotherapy in the Treatment of Patients with Silicotuberculosis in Hong Kong. Am Rev Resp Dis. 1991; 143(2):262–7. PMID: 1990938
13. Kim YH, Suh GY, Chung MP, Kim H, Kwon OJ, Lim SY, et al. Treatment of isoniazid-resistant pulmonary tuberculosis. BMC Infect Dis 2008; 8:6. doi: 10.1186/1471-2334-8-6 PMID: 18211720
14. Temple B, Ayakaka I, Ogwang S, Nabanjja H, Kayes S, Nakubulwa S, et al. Rate and amplification of drug resistance among previously-treated patients with tuberculosis in Kampala, Uganda. Clin Infect Dis 2008; 47(9):1126–34. doi: 10.1086/592252 PMID: 18808360
15. Yoshiyama T, Yanai H, Rhiengtong D, Palittapongarnpim P, Nampaisan O, Supawitkul S, et al. Development of acquired drug resistance in recurrent tuberculosis patients with various previous treatment outcomes. Int J Tuberc Lung Dis 2004; 8(1):31–8. PMID: 14974743
16. Algerian Working Group/British MRC. Short-course chemotherapy for pulmonary tuberculosis under routine programme conditions: a comparison of regimens at 28 and 36 weeks duration in Algeria. Tubercle. 1991; 72:88–100.
17. Lienhardt C, Cook SV, Burgos M, Yorke-Edwards V, Rigouts L, Anyo G, et al. Efficacy and safety of a 4-drug fixed-dose combination regimen compared with separate drugs for treatment of pulmonary tuberculosis: the Study C randomized controlled trial. JAMA. 2011; 305(14):1415–23. doi: 10.1001/jama.2011.436 PMID: 21486974
18. Tuberculosis Research Centre ICoMR. A controlled clinical trial of oral short course regimens in the treatment of sputum positive pulmonary tuberculosis. Int J Tuberc Lung Dis. 1(6):509–17.

19. Aung KJM, Declercq E, Ali MA, Naha S, Roy SCD, Taleb MA, et al. Extension of the intensive phase reduces relapse but not failure in a regimen with rifampicin throughout. Int J Tuberc Lung Dis 2012; 16 (4):455–61. doi: 10.5588/ijtld.11.0216 PMID: 22640511
20. Cox HS, Niemann S, Ismailov G, Doshetov D, Orozco JD, Blok L, et al. Risk of acquired drug resistance during short-course directly observed treatment of tuberculosis in an area with high levels of drug resistance. Clin Infect Dis 2007; 44(11):1421–7. PMID: 17479936
21. El Sahly HM, Teeter LD, Pawlak RR, Musser JM, Graviss EA. Drug-resistant tuberculosis: a disease of target populations in Houston, Texas. J Infect 2006; 53(1532–2742; 0163–4453; 1):5–11. PMID: 16310855
22. Murray J, Sonnenberg P, Shearer S, Godfrey-Faussett P. Drug-resistant pulmonary tuberculosis in a cohort of southern African goldminers with a high prevalence of HIV infection. S Afr Med J 2000; 90 (4):381–6. PMID: 10957924
23. Nettles RE, Mazo D, Alwood K, Gachuhi R, Maltas G, Wendel K, et al. Risk factors for relapse and acquired rifamycin resistance after directly observed tuberculosis treatment: a comparison by HIV serostatus and rifamycin use. Clin Infect Dis 2004; 38(5):731–6. PMID: 14986259
24. Pasipanodya JG, McIlleron H, Burger A, Wash PA, Smith P, Gumbo T. Serum Drug Concentrations Predictive of Pulmonary Tuberculosis Outcomes. J Infect Dis 2013; 208(9):1464–73. doi: 10.1093/infdis/jit352 PMID: 23901086
25. Chien JY, Lai CC, Tan CK, Chien ST, Yu CJ, Hsueh PR. Decline in rates of acquired multidrug-resistant tuberculosis after implementation of the directly observed therapy, short course (DOTS) and DOTS-Plus programmes in Taiwan. J Antimicrob Chemother. 2013; 68(8):1910–6. doi: 10.1093/jac/dkt103 PMID: 23580558
26. Driver CR, Munsiff SS, Li J, Kundamal N, Osahan SS. Relapse in persons treated for drug-susceptible tuberculosis in a population with high coinfection with human immunodeficiency virus in New York City. Clin Infect Dis 2001; 33(10):1762–9. PMID: 11595988
27. Gelmanova IY, Keshavjee S, Golubchikova VT, Berezina VI, Strelis AK, Yanova GV, et al. Barriers to successful tuberculosis treatment in Tomsk, Russian Federation: non-adherence, default and the acquisition of multidrug resistance. Bull World Health Organ 2007; 85(9):703–11. PMID: 18026627
28. Jasmer RM, Seaman CB, Gonzalez LC, Kawamura LM, Osmond DH, Daley CL. Tuberculosis treatment outcomes: directly observed therapy compared with self-administered therapy. Am J Respir Crit Care Med 2004; 170(1073–449; 1073–449; 5):561–6. PMID: 15184210
29. Li J, Munsiff SS, Driver CR, Sackoff J. Relapse and acquired rifampin resistance in HIV-infected patients with tuberculosis treated with rifampin- or rifabutin-based regimens in New York City, 1997–2000. Clin Infect Dis 2005; 41(1):83–91. PMID: 15937767
30. Moulding TS, Le HQ, Rikleen D, Davidson P. Preventing drug-resistant tuberculosis with a fixed dose combination of isoniazid and rifampin. Int J Tuberc Lung Dis 2004; 8(6):743–8. PMID: 15182145
31. Porco TC, Oh P, Flood JM. Anti-tuberculosis drug resistance acquired during treatment: an analysis of cases reported in California, 1994–2006. Clin Infect Dis 56(6):761–9. doi: 10.1093/cid/cis989 PMID: 23223590
32. Quy HT, Lan NT, Borgdorff MW, Grosset J, Linh PD, Tung LB, et al. Drug resistance among failure and relapse cases of tuberculosis: is the standard re-treatment regimen adequate? Int J Tuberc Lung Dis 2003; 7(7):631–6. PMID: 12870683
33. Seung KJ, Gelmanova IE, Peremitin GG, Golubchikova VT, Pavlova VE, Sirotkina OB, et al. The effect of initial drug resistance on treatment response and acquired drug resistance during standardized short-course chemotherapy for tuberculosis. Clin Infect Dis 2004; 39(9):1321–8. PMID: 15494909
34. Spellman CW, Matty KJ, Weis SE. A survey of drug-resistant *Mycobacterium tuberculosis* and its relationship to HIV infection. AIDS 1998; 12(2):191–5. PMID: 9468368
35. Weis SE, Slocum PC, Blais FX, King B, Nunn M, Matney GB, et al. The effect of directly observed therapy on the rates of drug resistance and relapse in tuberculosis. N Engl J Med 1994; 330(0028–4793; 0028–4793; 17):1179–84. PMID: 8139628
36. Yuen CM, Kurbatova EV, Click ES, Cavanaugh JS, Cegielski JP. 3871645; Association between *Mycobacterium tuberculosis* Complex Phylogenetic Lineage and Acquired Drug Resistance. PLoS One. 2013; 8(12):e83006. doi: 10.1371/journal.pone.0083006 PMID: 24376623
37. Bradford WZ, Martin JN, Reingold AL, Schecter GF, Hopewell PC, Small PM. The changing epidemiology of acquired drug-resistant tuberculosis in San Francisco, USA. Lancet. 1996; 348 (9032):928–31. PMID: 8843813
38. Munsiff SS, Joseph S, Ebrahimzadeh A, Frieden TR. Rifampin-monoresistant tuberculosis in New York City, 1993–1994. Clin Infect Dis 1997; 25(6):1465–7. PMID: 9431396

39. Weiner M, Benator D, Burman W, Peloquin CA, Khan A, Vernon A, et al. Association between acquired rifamycin resistance and the pharmacokinetics of rifabutin and isoniazid among patients with HIV and tuberculosis. Clin Infect Dis 2005; 40(10):1481–91. PMID: 15844071

40. Lew W, Pai M, Oxlade O, Martin D, Menzies D. Initial drug resistance and tuberculosis treatment outcomes: systematic review and meta-analysis. Ann Intern Med 2008; 149(2):123–34. PMID: 18626051

41. Menzies D, Benedetti A, Paydar A, Royce S, Pai M, Burman W, et al. Standardized treatment of active tuberculosis in patients with previous treatment and/or with mono-resistance to isoniazid: A systematic review and meta-analysis. PLoS Med 2009 6 (9).:e1000150. PMID: 20101802

42. Jacobson KR, Theron D, Victor TC, Streicher EM, Warren RM, Murray MB. Treatment outcomes of isoniazid-resistant tuberculosis patients, Western Cape Province, South Africa. Clin Infect Dis 2011; 53 (4):369–72 doi: 10.1093/cid/cir406 PMID: 21810750

43. Comas I, Borrell S, Roetzer A, Rose G, Malla B, Kato-Maeda M, et al. Whole-genome sequencing of rifampicin-resistant *Mycobacterium tuberculosis* strains identifies compensatory mutations in RNA polymerase genes. Nature Genetics. 2011; 44(1):106–10. doi: 10.1038/ng.1038 PMID: 22179134

44. Fenner L, Egger M, Bodmer T, Furrer H, Ballif M, Battegay M, et al. HIV Infection Disrupts the Sympatric Host-Pathogen Relationship in Human Tuberculosis. PLoS Genetics. 2013; 9(3):e1003318. doi: 10.1371/journal.pgen.1003318 PMID: 23505379

45. Strauss OJ, Warren RM, Jordaan A, Streicher EM, Hanekom M, Falmer AA, et al. Spread of a low-fitness drug-resistant *Mycobacterium tuberculosis* strain in a setting of high human immunodeficiency virus prevalence. J Clin Microbiol 2008; 46(4):1514–6. doi: 10.1128/JCM.01938-07 PMID: 18272712

46. Chideya S, Winston CA, Peloquin CA, Bradford WZ, Hopewell PC, Wells CD, et al. Isoniazid, rifampin, ethambutol, and pyrazinamide pharmacokinetics and treatment outcomes among a predominantly HIV-infected cohort of adults with tuberculosis from Botswana. Clin Infect Dis 2009; 48(12):1685–94. doi: 10.1086/599040 PMID: 19432554

47. Gumbo T, Louie A, Deziel MR, Liu W, Parsons LM, Salfinger M, et al. Concentration-dependent *Mycobacterium tuberculosis* killing and prevention of resistance by rifampin. Antimicrob Agents and Chemother 2007; 51(11):3781–8.

48. Pasipanodya J, Gumbo T. An oracle: antituberculosis pharmacokinetics-pharmacodynamics, clinical correlation, and clinical trial simulations to predict the future. Antimicrob Agents and Chemother 2011; 55(1):24–34.

49. Pasipanodya JG, Srivastava S, Gumbo T. Meta-analysis of clinical studies supports the pharmacokinetic variability hypothesis for acquired drug resistance and failure of antituberculosis therapy. Clin Infect Dis 2012; 55(2):169–77. doi: 10.1093/cid/cis353 PMID: 22467670

50. Ford CB, Shah RR, Maeda MK, Gagneux S, Murray MB, Cohen T, et al. *Mycobacterium tuberculosis* mutation rate estimates from different lineages predict substantial differences in the emergence of drug-resistant tuberculosis. Nat Genet 2013; 45(7):784–90. doi: 10.1038/ng.2656 PMID: 23749189

51. Borrell S, Gagneux S. Strain diversity, epistasis and the evolution of drug resistance in *Mycobacterium tuberculosis*. Clin Microbiol Infect 2011; 17(1469–0691; 1198–743; 6):815–20. doi: 10.1111/j.1469-0691.2011.03556.x PMID: 21682802

52. Pasipanodya JG, Gumbo T. A meta-analysis of self-administered versus directly observed therapy effect on microbiologic failure, relapse, and acquired drug resistance in tuberculosis patients. Clin Infect Dis 2013 57(1):21–31. doi: 10.1093/cid/cit167 PMID: 23487389

53. Ioannidis JP, Patsopoulos NA, Evangelou E. Uncertainty in heterogeneity estimates in meta-analyses. BMJ 2007 3; 335(7626):914–6. PMID: 17974687

54. Kontopantelis E, Springate DA, Reeves D. A re-analysis of the Cochrane Library data: the dangers of unobserved heterogeneity in meta-analyses.PLoS One 2013 Jul 26; 8(7):e69930. doi: 10.1371/journal.pone.0069930 PMID: 23922860

55. Albanna AS, Smith BM, Cowan D, Menzies D. Fixed dose combination anti-tuberculosis therapy: a systematic review and meta-analysis. Eur Resp J 2013 42 (3):721–322.

56. Menzies D, Benedetti A, Paydar A, Martin I, Royce S, Pai M, et al. Effect of duration and intermittency of rifampin on tuberculosis treatment outcomes: a systematic review and meta-analysis. PLoS Med. 2009; 6(9):e1000146. doi: 10.1371/journal.pmed.1000146 PMID: 19753109
